# Development of a 1:1-binding biparatopic anti-TNFR2 antagonist by reducing signaling activity through epitope selection

**DOI:** 10.1038/s42003-023-05326-8

**Published:** 2023-09-27

**Authors:** Hiroki Akiba, Junso Fujita, Tomoko Ise, Kentaro Nishiyama, Tomoko Miyata, Takayuki Kato, Keiichi Namba, Hiroaki Ohno, Haruhiko Kamada, Satoshi Nagata, Kouhei Tsumoto

**Affiliations:** 1 Graduate School of Pharmaceutical Sciences, Kyoto University, Sakyo-ku, Kyoto 606-8501 Japan; 2 Center for Drug Design Research, National Institutes of Biomedical Innovation, Health and Nutrition, Ibaraki, Osaka 562-0011 Japan; 3 Graduate School of Frontier Biosciences, Osaka University, Suita, Osaka 565-0871 Japan; 4 JEOL YOKOGUSHI Research Alliance Laboratories, Osaka University, Suita, Osaka 565-0871 Japan; 5 Graduate School of Pharmaceutical Sciences, Osaka University, Suita, Osaka 565-0871 Japan; 6 Institute of Protein Research, Osaka University, Suita, Osaka 565-0871 Japan; 7 RIKEN SPring-8 Center, Suita, Osaka 565-0871 Japan; 8 School of Engineering, The University of Tokyo, Bunkyo-ku, Tokyo 113-8656 Japan; 9 Institute of Medical Sciences, The University of Tokyo, Minato-ku, Tokyo 108-8639 Japan

**Keywords:** Antibody therapy, Protein design, Biophysical chemistry, Cryoelectron microscopy

## Abstract

Conventional bivalent antibodies against cell surface receptors often initiate unwanted signal transduction by crosslinking two antigen molecules. Biparatopic antibodies (BpAbs) bind to two different epitopes on the same antigen, thus altering crosslinking ability. In this study, we develop BpAbs against tumor necrosis factor receptor 2 (TNFR2), which is an attractive immune checkpoint target. Using different pairs of antibody variable regions specific to topographically distinct TNFR2 epitopes, we successfully regulate the size of BpAb–TNFR2 immunocomplexes to result in controlled agonistic activities. Our series of results indicate that the relative positions of the two epitopes recognized by the BpAb are critical for controlling its signaling activity. One particular antagonist, Bp109-92, binds TNFR2 in a 1:1 manner without unwanted signal transduction, and its structural basis is determined using cryo-electron microscopy. This antagonist suppresses the proliferation of regulatory T cells expressing TNFR2. Therefore, the BpAb format would be useful in designing specific and distinct antibody functions.

## Abstract (web summary)

Biparatopic antibodies binding to two different epitopes of tumor necrosis factor receptor 2 (TNFR2) are developed, and one of them binds TNFR2 in a 1:1 manner, functions as an antagonist without transducing intracellular signal.

## Introduction

Antibodies are widely harnessed as therapeutic agents. Reflecting their natural structure, most antibody therapeutics are conventional immunoglobulin G (cIgG) which have two variable regions for antigen binding. In addition to the high affinity of the single variable fragment (Fv), bivalency provides strong avidity binding to antigens, enhancing therapeutic efficacy.

**Fig. 1:**
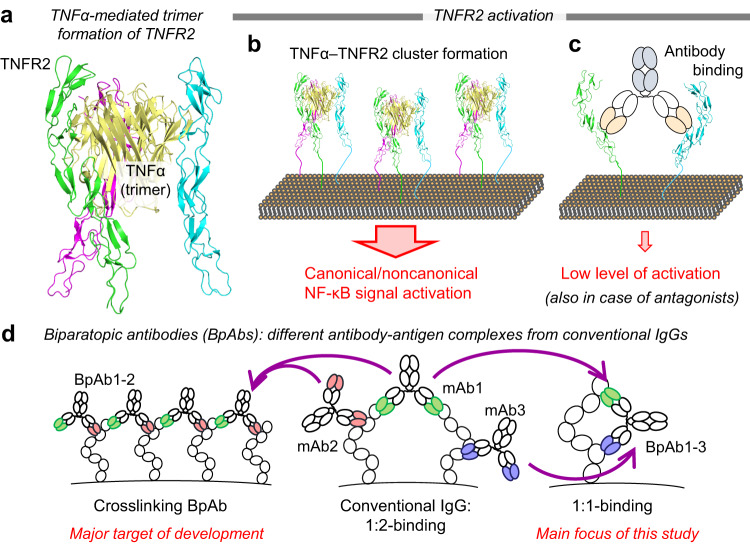
Activation of TNFR2 and biparatopic antibodies (BpAbs). **a** TNFR2 trimer is formed by interacting with TNFα (PDB entry: 3ALQ). **b** Trimer formation induces TNFR2 cluster formation, activating NF-κB signaling pathways. **c** Conventional antibodies, including antagonists, bind two TNFR2 molecules and may activate low levels of signaling. **d** Conventional IgG antibody binds the antigen in a 1:2 manner, whereas BpAbs, including the crosslinking type of BpAbs (BpAb1-2) and 1:1-binding BpAbs (BpAb1-3), involves a different topology of antibody-antigen complexes compared to conventional IgGs.

The present study focuses on tumor necrosis factor receptor 2 (TNFR2), a member of the tumor necrosis factor receptor superfamily (TNFRSF), as a promising target for antibody-based therapies due to its potential for effective treatment outcomes. TNFR2 clusters on the cellular membrane and is activated through their interactions with specific membrane-bound ligands^1–3^. TNFR2 aggregates upon binding to trivalent tumor necrosis factor-alpha (TNFα) (Fig. 1a, b)^1–4^. Aggregated TNFR2 increases the density of receptor-associated intracellular proteins, triggering the activation of downstream canonical and noncanonical NF-κB pathways^1,5,6^. TNFR2 is primarily expressed in a subset of T cells, including regulatory T cells (T_regs_)^7–10^, and TNFR2-mediated intracellular signaling expands T_regs_, inducing cancer cell proliferation. Therefore, anti-TNFR2 antagonists are promising as novel immune checkpoint inhibitors to enhance the antitumor immune response in cancer patients^9–13^. In contrast, anti-TNFR2 signal-inducing antibodies (agonists) trigger T_reg_ expansion in autoimmunity, organ transplant rejection, and graft-versus-host disease^9,10,14–16^. Therefore, the expansion of T_regs_ via TNFR2 activation is a promising therapeutic strategy.

Bivalency of cIgG can produce dimer formation-related unwanted cellular responses^17,18^. Similar to the natural TNFα ligand (Fig. 1b), anti-TNFR2 cIgGs may induce TNFR2 clustering by bivalent binding (Fig. 1c). Thus, antagonistic cIgGs may also induce signal transduction to also function as an agonist. TNFR1, a member of TNFRSF, is similarly activated by TNFα-mediated cluster formation. However, a potent TNFR1 antagonist antibody was reported to show adverse effects in humans due to its bivalency-dependent signaling activity^18,19^. Therefore, the agonistic activity of antagonist antibodies, irrespective of the mechanism of antagonism, needs to be minimized to broaden their clinical application potential. A homobivalent, antagonistic cIgG against TNFR2 was reported to require a specialized binding mode related to specific antigen-binding sites (epitopes) to prevent functional clustering^11^. However, this binding mode depends on empirical functional screening, thus hindering the design of an effective antagonist.

In this study, we developed biparatopic antibodies (BpAbs) against TNFR2 to enable an effective antagonist design. Here, an antagonist showed reduced TNFR2 signaling as the biological function, especially in the presence of TNFα. BpAb is an engineered bispecific antibody possessing two different antibody Fv that bind to different epitopes on the same antigen (Fig. 1d)^20,21^. By characterizing multiple bivalent IgG-like BpAbs targeting different topographical TNFR2 epitopes, we demonstrated that variable levels of agonistic activities were induced by different bivalent BpAbs, including BpAbs with no agonistic activity. Agonist potency depended on the ability of cIgGs and BpAbs to form clustered immunocomplexes with the antigen. Using physicochemical characterization and cryo-electron microscopy (cryo-EM), we demonstrated the binding of one potential antagonist, Bp109-92, to TNFR2 in a 1:1 manner (Fig. 1d, right). We showed that the functional activities of the engineered BpAbs are predictable based on the relative positions of the two epitopes. Thus, our findings lead to a fine-tuned design of TNFR2 agonists and antagonists based on BpAbs.

## Results

### Comprehensive production and biological activities of biparatopic antibodies

**Fig. 2:**
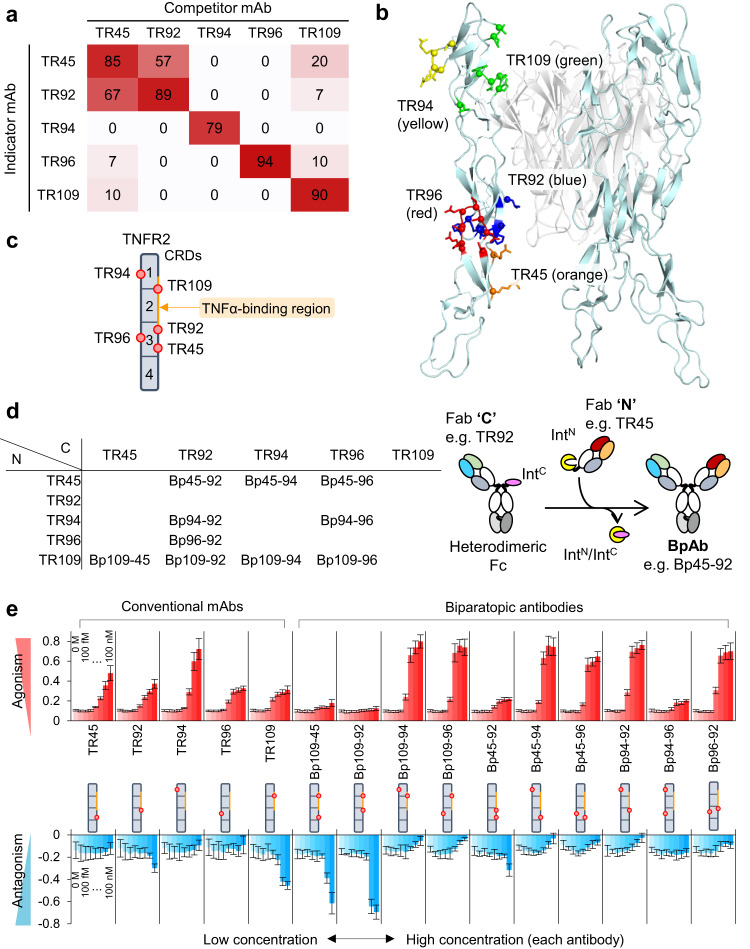
Monoclonal antibodies and biparatopic antibodies against TNFR2. **a** Topographical epitope mapping of the anti-TNFR2 mAbs by mutual competition analysis. Strengths of competition are shown as percentages in each box. **b** Epitopes of the selected monoclonal antibodies identified using the mutants. Cyan, TNFR2; white, TNFα (PDB entry: 3ALQ). **c** Schematic presentation of the epitopes (red circles). Cysteine-rich domains (CRDs) are separated by lines. **d** Produced BpAbs. Fab ‘**N**’ (row) and ‘**C**’ (column) were fused with Int^N^ or Int^C^-Fc, respectively, and were subjected to intein-mediated protein trans-splicing to produce a BpAb^38^. **e** Agonistic (upper) and antagonistic (lower) activity of conventional and biparatopic antibodies, monitored using a reporter cell line. Values are shown with the standard deviation of five independent experiments standardized by the reporter activity of 0 and 50 ng/mL TNFα.

We identified five anti-TNFR2 monoclonal antibodies (mAbs): TR45, TR92, TR94, TR96, and TR109. Either antibody did not bind to TNFα (Fig. S1). The five mAbs were selected from an epitope-normalized antibody panel, which is a technology used to obtain a panel of a minimum number of antibodies whose epitope regions are comprehensively and evenly distributed over the entire accessible surface of a target antigen^22^. Competitive ELISA was used to confirm mAb binding to different epitopes (Fig. 2a). To identify epitope sites on the surface of TNFR2, we designed a series of TNFR2 mutants by peptide grafting (Fig. S2 and Supplementary Data 1). First, mutants were generated to reduce binding to human TNFR2. For each mutant, the respective region of human TNFR2 was substituted with that of mouse TNFR2 (human-to-mouse mutants). The extracellular region of TNFR2 contains four cysteine-rich domains (CRDs; CRD1, 2, 3, and 4; Fig. S2b). For the first set of mutants (*n* = 9), CRD1, 2, and 3 were split by half, and CRD4 and C- and N-terminal mutants were generated by amino acid substitutions within each encoded region (“Mut1” line of Fig. S2a,c). We produced nine additional mutants for detailed analysis (“Mut2” line in Fig. S2a, d). Epitope sites were identified by reduced antibody binding to cells transiently transfected with wild-type human TNFR2 or human-to-mouse mutants (referred to as e.g., mC1A), which was determined using a flow cytometer. We identified regions that conferred a total or partial binding loss (Fig. S2e and Fig. S3). As a complementary analysis, mouse-to-human mutants were produced by replacing the peptides in mouse TNFR2 with a human ortholog (referred to as e.g., hC1A). Binding was only observed when the identified epitope region was present (Figs. S2f, S4), and TR109 required both hC1A2 and hC2A2 regions for binding. This result was consistent with the finding of reduced binding analysis for human-to-mouse mutants. All five mAbs bound to different parts of the human TNFR2, consistent with competitive ELISA, and the binding sites revealed by multiple mutation studies were mapped (Fig. 2b, c). The epitopes of TR92 and TR109 overlapped with the TNFα-binding region. The epitope of TR45 was non-overlapping but on the same side as the TNFα-binding region, whereas those of TR94 and TR96 were located on the opposite side of the TNFα-binding region. Bivalent IgG-like BpAbs of the ten Fv combinations from the five mAbs were produced (Fig. 2d) and confirmed to bind to the same epitopes as the original cIgGs (Figs. S2f, S4).

Next, we used reporter cells to detect NFκB-dependent DNA transcription. TNFR2 expression of reporter cells was roughly equivalent to the highest level of the TNFα-stimulated peripheral blood mononuclear cells (PBMCs) which heterogeneously expressed TNFR2 (Figs. S5, S6). The agonistic and antagonistic activities of cIgGs and BpAbs were evaluated in the absence or presence of TNFα (Fig. 2e). The BpAb panel included the majority of six strong agonists with high maximum agonistic activity at low concentrations, compared with those of cIgGs. It should be noted that all five cIgGs were moderately agonistic, supposedly reflecting their bivalency. In contrast, BpAbs contained two antagonists (Bp109-45 and Bp109-92) with significantly reduced agonistic activity. Bp109-92 displayed a strong antagonistic effect against TNFα-dependent activity without any functional agonistic activity. Thus, we successfully produced an artificial antagonistic BpAb that does not occur naturally and has not been previously generated. These combined results suggest that BpAbs can be designed with or without desired functions using selected pairs of Fvs that recognize two non-overlapping epitopes.

### Binding analysis and the size of immunocomplexes

To examine the origin of their agonistic activities, we analyzed the concentration-dependent binding of cIgGs and BpAbs to reporter cells using flow cytometry. All cIgGs and BpAbs, except for TR94, showed typical sigmoidal curves, with apparent IC_50_ values of ~30 ng/mL (corresponding to 0.2 nM) (Fig. S7). The affinity of cIgGs and BpAbs for TNFR2 was also measured by surface plasmon resonance (SPR) using sTNFR2, digested from etanercept, as the antigen (Fig. S8). The large majority of BpAbs bound more strongly than cIgGs (*K*_D_ = 0.92 nM versus 7.7 nM on average; Fig. S9 and Table S1). In either experiment, the strength of the interaction by BpAbs did not correlate with maximum agonistic activity; however, the curved shape of cellular binding coincided with agonistic activity (Fig. S7). This phenomenon is in contrast with the observation of cIgGs, with the weakest binder (TR94) showing the strongest maximum agonistic activity, which was consistent with a recent report targeting other TNFRSF members^23^. The binding-activity relationship indicated that the higher-order structure of the immunocomplex was a key determinant of functional activity.

**Fig. 3:**
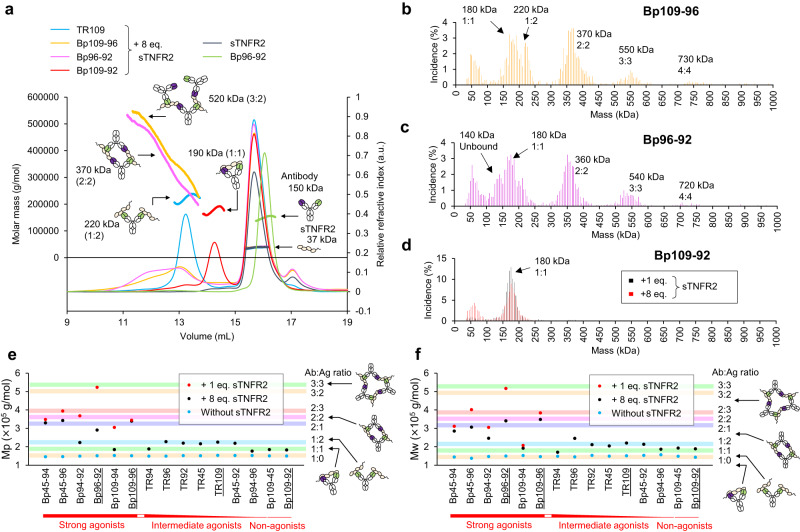
Size of the immunocomplexes formed by conventional and biparatopic antibodies. The size was analyzed by SEC-MALS (**a**, **e**, **f**) or mass photometry (**b**–**d**). **a** Selected chromatograms of SEC-MALS. Relative refractive index and molar mass are represented by thin and bold lines, respectively. **b**–**d** Relative frequency of the observed particles with their respective mass in mass photometry. Bp109-96 (**b**), Bp96-92 (**c**), or Bp109-92 (**d**) was mixed with 1 eq. or 8 eq. (only for Bp109-92) of sTNFR2. Peak molecular weight (Mp) (**e**) or weight-averaged molecular weight (Mw) (**f**) of the total fractions of immunocomplexes (eluted earlier than 14.8 mL). Chromatograms of underlined antibodies are shown in (**a**). Colored bands represent the estimated size of the immunocomplex with the expected structures in the right column.

Size-exclusion chromatography combined with multi-angle light scattering (SEC-MALS) was conducted to analyze the size of the antigen-antibody complexes. Chromatograms of TR109 and the three BpAbs using the variable regions of TR92, TR96, and TR109 are shown in Fig. 3a. sTNFR2 had a molar mass of 37 kDa, and Bp96-92 alone had a molar mass of 150 kDa (other cIgGs and BpAbs had the same size; Fig. S10). TR109 formed a homogeneous 1:2 antibody to the TNFR2 complex of 220 kDa in the presence of excess sTNFR2. Bp96-92 and Bp109-96 formed larger heterogeneous complexes, with a broad distribution of molar masses ranging from 200 to 500 kDa. These two agonistic BpAbs were capable of forming large bridging immunocomplexes. This is consistent with previous reports of BpAbs bridging multiple antigen molecules to function, including TNFRSF (Fig. 1d, left)^20,24–26^. In contrast, antagonist Bp109-92 formed a homogeneous 1:1 complex of 190 kDa in the presence of excess sTNFR2; thus, 1:1 complex formation was much more advantageous than 1:2 complex formation. The complex size was analyzed using mass photometry at the single-particle level and similar results were obtained. For Bp109-96 and Bp96-92, equimolar BpAb-antigen complexes up to an antibody to TNFR2 ratio of 4:4 were observed (Fig. 3b, c). In contrast, the antibody to TNFR2 1:1 complex was dominant in Bp109-92 (Fig. 3d). The observation of equimolar complexes indicated that the two variable regions of BpAb were both bound to TNFR2, and the observation by SEC-MALS was the average of mixed immunocomplexes at elution, reflecting their size distribution quantitatively.

Other cIgGs and BpAbs were also analyzed using SEC-MALS (Fig. 3e, f and Fig. S10). The six strong agonist BpAbs formed large immunocomplexes, whereas the immunocomplexes of the non-agonist BpAbs were smaller than those of cIgGs. As an exception, the poor binding ability of TR94 (Fig. S8) resulted in a smaller complex size. A similarly smaller immunocomplex was observed for Bp109-94 in the presence of excess sTNFR2, whereas a large immunocomplex was observed with an equimolar sTNFR2 concentration (Fig. S10). The larger immunocomplex observed for the equimolar mixture was shared among agonistic bridging BpAbs, possibly due to the disadvantage in 1:2 complex formation (Fig. S11). In summary, cIgGs and BpAbs were categorized into three classes: (1) large immunocomplexes formed by strong agonist BpAbs, (2) 1:2 complex of antibody to TNFR2 by moderate agonists, and (3) 1:1 immunocomplexes without agonistic activity including antagonistic BpAbs. A simple relationship was observed over a broad concentration range.

The crosslinking ability of antibodies was further evaluated by their potential for multivalent binding in SPR. Maltose-binding protein (MBP) fusion of TNFR2 (TNFR2-MBP, Fig. S8) was captured by anti-MBP antibodies at four different levels, and the binding kinetics of cIgGs and BpAbs were analyzed (Figs. S12, S13, and Table S2). Informative analysis was not successful for TR94 due to its weak affinity. In addition, the slow dissociation of TR96 and BpAbs bearing the Fv prevented the determination of the dissociation rate at the high capture density of TNFR2-MBP (Table S2). Despite these challenges, the curve shapes in Fig. S12 provide information on the different binding kinetics dependent on the capture density. Most cIgGs and BpAbs showed a slower dissociation rate by higher capture levels. In contrast, Bp109-92 showed minimum dependency on the capture density. This result strongly indicated the absence of a crosslinking binding pattern (1:2 or larger) by Bp109-92.

Bp45-94 (agonist) and Bp109-92 (antagonist) in complex with sTNFR2 were analyzed using negative-stain EM. In both cases, sTNFR2 was not visible, owing to its small size and low resolution. Bp45-94 showed a variety of structures up to a complex comprising three BpAb molecules; however, most particles were monomers or dimers (Fig. S14a). Monomers were presumably unbound or bound by one or two sTNFR2 molecules but were not distinguishable. The dominant larger complex was a dimer, consistent with the observations by SEC-MALS. However, mainly monomers were observed for Bp109-92, even in the presence of sTNFR2 (Fig. S14b). When the Bp109-92 F(ab′)_2_–sTNFR2 complex was analyzed by enzymatic removal of Fc, a parallel arrangement of two antigen-binding fragments (Fabs) was observed (Fig. S14c). This contrasts with the random orientation of Bp109-92 F(ab′)_2_ in the absence of sTNFR2 (Fig. S14d).

### Precise characterization of the antagonist, Bp109-92

**Fig. 4:**
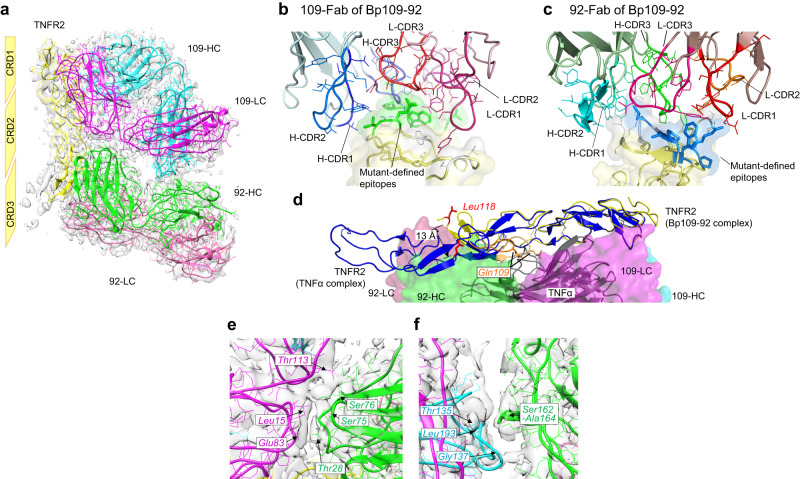
Cryo-EM structure of Bp109-92 in complex with TNFR2. **a** Overall view of the cryo-EM structure. 109-HC/LC and 92-HC/LC are the heavy/light chains from TR109 and TR92, respectively. **b**, **c** Enlarged view of the epitope site of 109-Fab (**b**) and 92-Fab (**c**) observed for the complex with BpAb. Each complementarity-determining region (CDR) loop is colored separately. Mutant-defined epitope amino acids are colored green (**b**) or blue (**c**), while interacting amino acids not clearly defined as the epitope residue are colored white. **d** Structural difference of TNFR2 observed in complex with Bp109-92 (yellow) and with TNFα (blue) (PDB entry: 3ALQ). 109-Fab and 92-Fab are shown with the van der Waals surface in the same color as **a**, while TNFα is shown with a cartoon in gray. Leu118 and C3A2 regions of the two TNFR2 structures are colored red and orange, respectively. **e**, **f** Fab-Fab contacts inside Bp109-92 in complex with TNFR2. **e** Interface between 109-LC and 92-HC. **f** Interface between 109-HC and 92-HC.

After several rounds of optimization, the three-dimensional structure of the Bp109-92–TNFR2 1:1 complex was determined at a resolution of 3.63 Å by cryo-EM single-particle image analysis using TNFR2-MBP (Table 1, Figs. S15, S16). In the complex structure (Fig. 4a), Fabs from both TR92 and TR109 (92-Fab and 109-Fab) were in a parallel arrangement, as observed by negative-stain EM (Fig. S14d), and biparatopic binding involving the simultaneous binding of both variable regions was observed. The epitope locations of the two mAbs determined by mutagenesis matched the Fab–TNFR2 interfaces (Fig. 4b, c), which was shared by the TNFR2–TNFα interface in the reported crystal structure (Protein Data Bank [PDB] entry:3ALQ) (Fig. 4d)^4^. The overlap of the Bp109-92 and TNFα-binding sites of TNFR2 indicated the mechanism of antagonistic activity directly derived from ligand blocking. This was further supported by competitive binding analysis in SPR (Fig. S17).

**Table 1:** Cryo-EM data collection and image processing of Bp109-92–TNFR2-MBP complex.

	Bp109-92–TNFR2-MBP complex (EMDB-34871) (PDB 8HLB)
Data collection and processing	
Magnification	60,000
Voltage (kV)	300
Electron exposure (e^−^/Å^2^)	60
Defocus range (μm)	−0.5 to −2.0
Pixel size (Å)	1.088
Symmetry imposed	C1
Micrographs used (no.)	4,160
Initial particle images (no.)	2,218,071
Final particle images (no.)	100,391
Map resolution (Å)	3.63
FSC threshold	0.143
Refinement	
Initial model used (PDB code)	3ALQ
Model resolution (Å)	3.5/3.6/4.0
FSC threshold	0/0.143/0.5
Model vs. Data CC (mask)	0.75
(volume)	0.73
Model composition	
Non-hydrogen atoms	7464
Protein residues	977
Ligands	0
R.m.s. deviations	
Bond lengths (Å)	0.006
Bond angles (°)	0.761
Validation	
MolProbity score	2.05
Clashscore	10.35
Poor rotamers (%)	0
Ramachandran plot	
Favored (%)	91.21
Allowed (%)	8.38
Outliers (%)	0.41

The density corresponding to Fc was not clearly observed. As for the antigen, TNFR2 possesses 4 CRDs as structural units in the extracellular region. The atomic model of TNFR2 was only partially built, ranging from cysteine-rich domain 1 (CRD1) to the middle of CRD3, corresponding to the C3A region in Fig. S2, although the density corresponding to CRD3 and CRD4 was weak (Fig. S18). CRD3 and CRD4 were oriented differently from the crystal structure of TNFR2 in the complex with TNFα (Fig. 4d and Fig. S18). The overall structures of 109-Fab and 92-Fab were similar (r.m.s.d. = 1.20 Å among 397 C_α_ atoms), but the complementarity-determining region (CDR) regions were significantly changed to interact with different regions of TNFR2 (Fig. S19). In the TNFR2–TNFα complex, Gln109 of TNFR2 interacts with TNFα, and the loop of the C3A2 region (orange in Fig. 4d) is pulled toward TNFα. In contrast, this loop partly protruded into a unique cleft between heavy chain CDR 1 (CDR-H1) and CDR-H3 of 92-Fab in the Bp109-92 complex (Fig. 4c) and pulled in the opposite direction. Leu118 at the C-terminus of C3A was shifted by 13 Å when the two TNFR2 structures were aligned by CRD1 and CRD2 (Fig. 4d). Flexibility of the CRDs of TNFR2 was indicated.

For Bp109-92 bound to TNFR2, contact between 109-Fab and 92-Fab was observed (Fig. 4e, f). This contact surface was formed by the heavy chain variable domain (VH) of 92-Fab entering the concave surface at the elbow region of 109-Fab and interacting with the linker between the variable and constant regions of the light chain. Although the resolution was not high enough to discuss the respective atomic-level contacts, this phenomenon may energetically contribute to the stabilization of the immunocomplex.

**Fig. 5:**
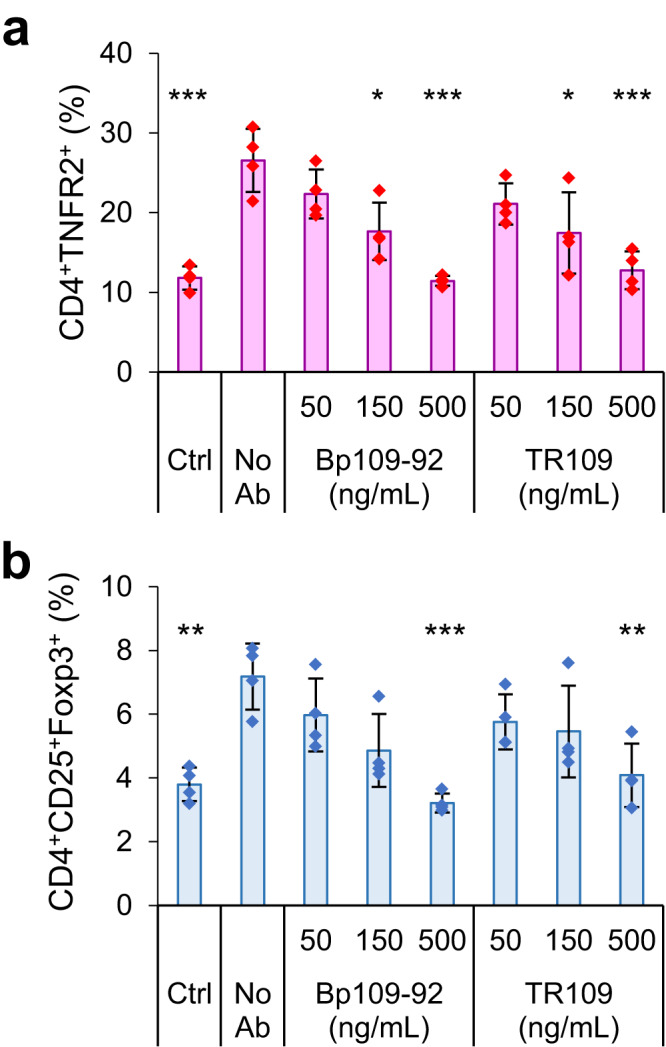
Suppression of TNFR2 activity in human T cells by the antagonists. The population of CD4^+^TNFR2^+^ cells (**a**) or CD4^+^CD25^+^Foxp3^+^ cells (**b**) among CD3^+^ cells in PBMC without treatment (Ctrl), in the presence of 50 ng/mL TNFα (No Ab), or in the presence of TNFα and antibodies. The values obtained in each experiment are shown as dots, and the average of the four independent experiments is shown with bars. Error bars represent the standard deviations. *Statistical significance in comparison with “No Ab”, Tukey’s test, *, *P* < 0.05; **, *P* < 0.005; ***, *P* < 0.001. See Table S3 for the adjusted *P*-values.

Finally, we used PBMCs to demonstrate the applicability of Bp109-92 in the inhibition of TNFα-dependent proliferation of T cells (Fig. S5 for the gating strategy). TNFα stimulation increased the population of TNFR2^+^ cells among CD3^+^ T cells (Fig. S20a). This population coincided with CD4^+^CD25^+^CD127^low^ cells, which contain Foxp3^+^ regulatory T cells (Fig. S20b). Both TR109 or Bp109-92 effectively reduced TNFR2^+^ cells in a concentration-dependent manner, thus acting as antagonists of TNFα-dependent proliferation (Fig. 5a, Table S3). The population of TNFR2^+^ cells increased from 12% to 27% after TNFα treatment; however, 500 ng/mL Bp109-92 or TR109 suppressed the population to 11% and 13%, respectively. A similar reduction was observed for Foxp3^+^ cells, which were also increased by TNFα (Fig. 5b, Table S3). The population of Foxp3^+^ cells increased from 3.8% to 7.2% with TNFα treatment, which was reduced to 3.2% and 4.1% in the presence of 500 ng/mL Bp109-92 or TR109, respectively. Thus, both Bp109-92 and TR109 were effective antagonists, and the antagonistic activity of Bp109-92 was greater than that of TR109. We further analyzed the antagonistic activity of F(ab′)_2_ of Bp109-92 and TR109 (Fig. S21, Table S4). Both the populations of CD3^+^CD4^+^CD25^+^Foxp3^+^ cells and CD3^+^CD4^+^TNFR2^+^ cells among CD3^+^ cells were not significantly affected by Fc removal. Slight differences were observed in the CD3^+^CD4^+^CD25^+^Foxp3^+^ population between Bp109-92 F(ab′)_2_ and TR109 cIgG, implicating the possibility of a small level of Fc receptor-mediated agonistic effect which can be amplified in the presence of cIgG-mediated bivalent binding (Fig. 1c). These results supported the advantage of the 1:1-binding Bp109-92 as an antagonist.

## Discussion

Recent advances in antibody engineering have paved the way to regulate the stoichiometry and size of complexes between antigen and antibody molecules^21^. As one of the most promising techniques, artificial BpAbs have overcome the limitations of cIgGs by releasing from the restriction of homobivalent interaction. Manipulating the size of the complex enables BpAbs against cell surface receptors to control intracellular signaling on a mechanistic basis^20^. Despite its importance, rational selection of appropriate pairs of Fvs for designing desired functional BpAbs has not yet been established, except for a small number of examples^27,28^. In the present study, we demonstrated that the signal transduction activities of anti-TNFR2 BpAbs were precisely controlled by the epitope locations used in bivalent BpAbs. Clear switching of BpAb agonism and antagonism was not achieved in the cIgG format. A bivalent IgG-like bispecific format has enabled various signaling activities.

Notably, 1:1 complex formation by Bp109-92 resulted in the nearly complete elimination of the agonistic activity and maximized the antagonistic function. This type of 1:1 binding complex is similarly prepared using single Fab or Fv-based formats^19,29^, including a monovalent antagonist against TNFR1 (Atrosimab) sharing a homologous epitope with TR109 in our study (Fig. S22)^19,30^. However, a single Fab or Fv lacks binding avidity, and its binding activity is reduced. In the above case, Atrosimab showed reduced binding to TNFR1 (three-fold reduction) to the original bivalent cIgG, although artificial affinity maturation was conducted^31^. Developers of Atrosimab favor the monovalent antibody to avoid homobivalency-dependent unwanted responses observed in early clinical trials^18^. In contrast, in our study, the affinity of Bp109-92 for TNFR2 was 25- and 35-fold higher than parental cIgGs (Table S1), and strong binding, similar to that of the parental cIgGs with avidity (Fig. S7 and Table S2), was observed. Increased apparent binding affinity of designed BpAbs is common^20^. BpAbs binding to trimeric viral antigens in a 1:1 manner are estimated in several examples^32,33^; however, 1:1 binding is not considered biologically functional. We provide the evidence that the exclusive formation of a specific 1:1 complex is biologically significant. Thus, we showed that limiting immunocomplexes to a 1:1 ratio with cell surface proteins can be an effective therapeutic approach.

**Fig. 6:**
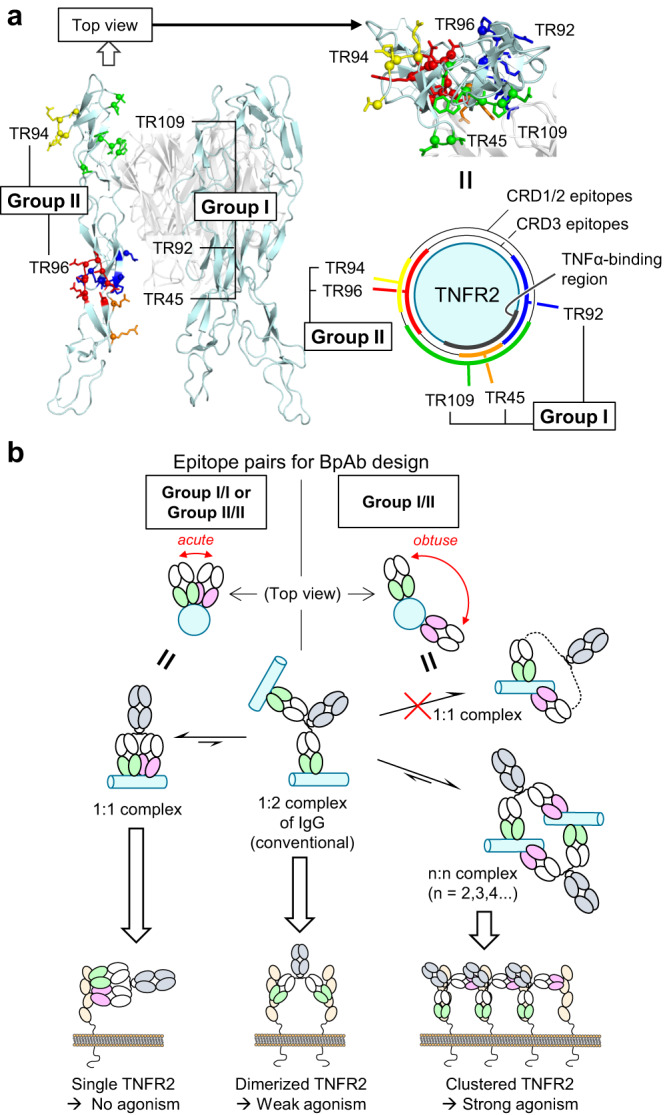
Epitope selection to control agonistic activity. **a** Two groups of epitopes for designing biparatopic antibodies (BpAbs). **b** Mechanism of control over agonistic activity through crosslinking activity.

Our study also leads to a rational design of bioactive BpAbs targeting TNFRSF proteins. Based on epitope analysis, we found that agonistic activity dependent on complex size can be simply controlled by the relative positioning of the two variable epitope locations used for BpAbs. Schematically, the CRDs of TNFR2 constituted a cylinder-like structure, and the epitopes of the five mAbs were mapped onto the cylindrical model viewed from the top (Fig. 6a). The epitopes of the five mAbs are grouped into group I consisting of the TR109, TR92, and TR45 epitopes on the same side as the TNFα-binding region of TNFR2, and group II consisting of the other two epitopes. In the top view of TNFR2, epitopes in the same group were in proximity, and the two Fabs formed acute angles with each other (Fig. 6b, left), as observed in the cryo-EM structure of Bp109-92 (Fig. 4a). BpAbs designed by selecting two epitopes in the same group would be advantageous for forming a 1:1 complex of antibody to an antigen unless a competition occurs. In contrast, when epitopes from two different groups are not in close proximity (Fig. 6b, right), 1:1 complex formation is impossible and large n:n complexes (*n* = 2,3,4 and higher) would dominate. Once the immunocomplex is formed, 1:1 complexes do not induce association-dependent signal transduction (non-agonist), 1:2 complexes formed by cIgGs work as weak agonists, and large n:n complex formation induces strong signal transduction (strong agonist). Antagonists are selected among 1:1-binders. As observed in the cryo-EM structure of the Bp109-92–TNFR2 complex, the CRDs of TNFR2 may have some flexibility; thus, both 1:1 and large n:n complex formation would be possible without steric or torsional hindrance. The epitope dependency of the agonistic activity of BpAbs against TNFR2 in our study could guide the development of both effective agonists and antagonists against other members of TNFRSF proteins that share similar activation mechanisms. As tetravalent BpAbs effectively agonize OX40^25^, the design contributes to the production of molecular therapeutics for immunology and oncology^3,17,18,34^.

This study has some limitations. The success of the BpAb design observed in this study may be attributed to its focus on developing a ligand-blocking antagonist with a simple mechanism of action. However, recombinant protein-based analysis may not be sufficient for analyzing the mechanism of action of effective agonists, which may depend on the cell surface density of the receptors and the signaling mechanism. For these applications, further analysis of cellular events may be required. Furthermore, the design also depends on the mechanism of TNFRSF activation. For example, receptor tyrosine kinases are activated by phosphorylation, which is activated by the formation of a specific dimerized structure. In these cases, specific BpAbs or alternative scaffolds are designed to lock them into inactive structures^35–37^, and a more precise design may be required.

In conclusion, we successfully developed various functional BpAbs and elucidated a simple mechanism for regulating the agonistic activity by comprehensively screening bivalent BpAbs against TNFR2. In particular, we developed Bp109-92, which had strong antagonistic activity without any agonistic activity. It was unique as it only had a specific 1:1-binding mode. Further research is required to assess the clinical efficacy of this antagonistic type against TNFR2. Nevertheless, this study provides a useful framework for designing BpAbs with the desired biological properties. The possible limitation of this design lies in the structure of the target, as 1:1-binding requires a large, exposed surface of the target protein to cover the two epitopes, at an appropriate distance. Nonetheless, we believe that BpAbs with similar designs against TNFRSF and other cell surface receptors may expand the potential of antibody therapeutics.

## Materials and methods

### Cell culture

Ramos-Blue cells (InvivoGen, San Diego, CA, USA) and human TNFR2-expressing Ramos-Blue cells were cultured in IMDM supplemented with 2 mM glutamine and 10% fetal bovine serum (FBS)^38^. The TNFR2-expressing cell line was developed in-house previously^38^ and is not commercially available. HEK293T cells were obtained from American Type Culture Collection (ATCC) and cultured in D-MEM supplemented with 10% FBS. Human PBMCs, obtained from healthy Community Blood Bank (Sioux Falls, SD, USA) donors, were cultured in RPMI1640 supplemented with 2 mM glutamine and 10% FBS.

### Competitive binding analysis

The topographical relationships between the binding of the five anti-TNFR2 mAbs (TR45, TR92, TR94, TR96, and TR109) were analyzed by the mutual competition of all possible mAb pairs (5 × 5 = 25) using a label-free competitive ELISA^39,40^. Microtiter plates were coated with goat anti-mouse IgG Fc and each indicator mAb#1 (2 μg/mL) was captured. An excess amount of competitor mAb#2 (5 μg/mL in blocking buffer) mixed with 20 ng/mL of TNFR2-rabbit Fc fusion protein (TNFR2-rFc)^38^ was incubated overnight at 4 °C in a separate tube. The plates were washed twice and the mAb#2–TNFR2-rFc solution was added to each well of the coated plates. Immune complexes captured on the plates were probed with alkaline phosphatase-conjugated goat anti-rabbit IgG (1/4000 dilution). The binding of mAb#1 to the mAb#2–TNFR2-rFc immune complex was determined as the percentage of binding to TNFR2-rFc. No competition (less than 0) was indicated as 0.

### Cloning, expression, and purification of chimeric recombinant antibodies

Chimeric antibodies with human IgG1 and Igκ constant regions were produced by cloning DNA and encoding the variable regions of the heavy and light chains into pFUSE-CHIg-hG1 and pFUSE2-CLIg-hκ (InvivoGen), respectively. Recombinant chimeric antibodies were produced using the Expi293 Expression System (Thermo Fisher Scientific, Waltham, MA, USA), according to the manufacturer’s protocol. cIgGs were purified from the culture supernatant using a 1 mL HiTrap Protein A HP Column (Cytiva, Marlborough, MA, USA). BpAbs were produced by intein-mediated protein trans-splicing in a method described previously^38^. Briefly, Fab **‘N’** (Fig. 2d) was fused to the Cfa Int^N^- MBP, and Fab **‘C’** was fused to an Fc with a ‘knob’ mutation using the knobs-into-holes method, which was co-expressed with an MBP-Cfa Int^C^ fusion of Fc with “hole” mutation to produce a monovalent antibody. Both proteins were produced using the Expi293 Expression System, and were added to 2 mM Tris(2-carboxyethyl)phosphine (TCEP) and incubated at 37 °C for 2 h. Disulfide bonds were restored by 1 mM oxidized glutathione, and MBP-fused side products were removed using amylose resin (New England BioLabs, Ipswich, MA, USA). The BpAbs were purified in a Superdex 200 Increase 10/300 GL column (Cytiva). The antibodies were stored in phosphate-buffered saline (PBS, pH 7.4).

F(ab′)_2_ was prepared by incubating TR109 or Bp109-92 with IdeS, an Fc-specific protease. IdeS with a C-terminal hexahistidine tag in PBS (2 mg/mL)^41^ was added to the antibodies at 10% (w/w), and the solution was incubated at 37 °C for 1 h. Following incubation, IdeS was removed using a 0.5 mL cOmplete-HisTag Purification Resin (Roche Diagnostics, Basel, Switzerland) column. The column was washed with 2 mL of PBS. To remove unreacted antibodies and fused Fc, the flow-through and wash fractions were passed through a 0.5 mL rProtein A Sepharose Fast Flow (Cytiva) column. The column was washed with 2 mL of PBS. The F(ab′)_2_ proteins in the flow-through and wash fractions were purified on a Superdex 200 Increase 10/300 GL column.

### Preparation of recombinant TNFR2 proteins for physicochemical and structural analyses

sTNFR2 was obtained from the Fc-fused TNFR2 drug, etanercept (Pfizer, New York, NY, USA). IdeS was used to isolate the extracellular domains of TNFR2 from etanercept following the same protocol as producing F(ab′)_2_. The hinge-mediated dimer of extracellular domains of TNFR2, obtained in the flow-through and wash fractions from protein A affinity purification, was further purified using a HiLoad Superdex 200 16/600 column. To obtain monomeric sTNFR2 extracellular domains, dimeric TNFR2 was reduced using 50 mM 2-mercaptoethylamine at 37 °C for 2 h. The solution was dialyzed in PBS, containing 1 mM EDTA, for 4 h. Subsequently, the solution was dialyzed in PBS for 3 days, with the buffer exchanged daily. The produced sTNFR2 was purified on a HiLoad Superdex200 16/600 column, and after concentration on an ultrafiltration unit (Amicon-Ultra-15, 10 K, Merck KGaA, Darmstadt, Germany), size-exclusion chromatography was conducted using a Superdex 200 Increase 10/300 GL column to remove aggregated proteins.

The MBP fused to the four CRDs of TNFR2 (TNFR2-MBP) was cloned into pcDNA3.1. Protein was expressed using the Expi293 Expression System (Thermo Fisher Scientific). Six days post-transfection, the culture supernatant was dialyzed overnight against Buffer A (20 mM Tris, 300 mM NaCl, pH 8.0) containing 5 mM imidazole. Expressed proteins were captured on Ni-NTA Superflow (Qiagen, Hilden, Germany) equilibrated with Buffer A containing 5 mM imidazole. The resins were washed sequentially using Buffer A containing 5, 10, and 20 mM imidazole, and the protein was eluted using Buffer A containing 200 mM imidazole. The eluate was dialyzed against PBS and the final purification was conducted using a Superdex200 16/600 column.

### Surface plasmon resonance (SPR)

SPR measurements were conducted using a Biacore T200 instrument (Cytiva).

Interactions between antibodies or TNFR2-MBP with immobilized TNFα were analyzed in HBS-EP+ at a flow rate of 30 μL/min at 25 °C. TNFα (130 ng/mL in 10 mM sodium acetate, pH 5.5), was immobilized on a CM5 chip using Amine Coupling Kit (Cytiva) at a flow rate of 5 μL/min for 120 s to reach 140 RU. For antibody-binding analysis against TNFα, antibodies (10 nM or 100 nM) were flowed for 120 s of contact time followed by 600 s of dissociation. For the ligand-blocking experiment, the mixture of TNFR2-MBP (25 nM) and antibody (50 nM) was flowed for 120 s of contact time followed by 300 s of dissociation. TNFα was regenerated by 1 M Arg-HCl (pH 4.4) run for 15 s^42^.

Interactions between sTNFR2 with captured cIgGs or BpAbs were analyzed in PBS supplemented with 0.005% Tween 20 (pH 7.4) at a flow rate of 30 μL/min at 25 °C. The antibodies were captured on a CM5 chip immobilized with anti-human IgG Fc using a Human Antibody Capture Kit (Cytiva), according to the manufacturer’s protocol. The antibodies (1 μg/mL) were run for 120 s to capture ~400 RU. Analytes were used in a two-fold dilution series of 1.25–80 nM (TR94 cIgG), 0.156–10 nM (cIgG and BpAbs bearing TR96 variable region), 0.313–10 nM (Bp94-92 and Bp109-92), 0.625–20 nM (TR92 cIgG), or 0.625–40 nM (others). The contact and dissociation times for the antigen were 90 and 300 s, respectively, and the anti-human IgG Fc antibody was regenerated by 3 M MgCl_2_ run for 30 s. The kinetic parameters were obtained by 1:1 global kinetics fitting using Biacore T200 Evaluation Software.

Interactions between antibodies with captured TNFR2-MBP were analyzed in HBS-EP+ at 25 °C. Anti-MBP Monoclonal Antibody (1 mg/mL, New England BioLabs) was diluted to 1/20 by 10 mM sodium acetate, pH 4.5, and was immobilized on a CM5 chip using Amine Coupling Kit at a flow rate of 5 μL/min for 400 s to reach 6000 RU. TNFR2-MBP was captured on the sensor chip at a flow rate of 30 μL/min. To capture 12 RU or 45 RU, 5 nM solution was flowed for 8 s or 30 s, respectively. To capture 120 RU or 300 RU, 20 nM solution was flowed for 20 s or 60 s, respectively. Anti-TNFR2 cIgGs and BpAbs as analytes were used in a two-fold dilution series of 0.4–6.4 nM, and were run in single-cycle kinetics at a flow rate of 10 μL/min. The contact and dissociation times were 180 and 1200 s, respectively, and the anti-MBP antibody was regenerated by 3 M MgCl_2_ run for 30 s for three times of repetition. The kinetic parameters were obtained by 1:1 kinetics fitting using Biacore T200 Evaluation Software.

### Flow cytometry

The immunochemical reagents used were as follows: anti-human IgG, Fcγ-PE (1/200 dilution, #109-116-170; Jackson Immunoresearch, West Grove, PA, USA), CD3-BV711 (1/250 dilution, #300463; BioLegend, San Diego, CA, USA), CD4-BV510 (1/100 dilution, #344633; BioLegend), CD25-BV421 or CD25-BV605 (1/40 dilution, #302629 or #302631; BioLegend); CD127-PerCP-Cy5.5 (1/40 dilution, #560551; BD Biosciences, Franklin Lakes, NJ, USA), TNFR2-PE (1/70 or 1/100 dilution, #22235; R&D Systems, Minneapolis, MN, USA), and Foxp3-AlexaFluor 647 (1/100 dilution, #320213; BioLegend). Cells were treated with PBS containing 0.2% sodium azide and 5% FBS. For the antibody-binding analysis of TNFR2-expressing Ramos-Blue and HEK293T cells, primary antibodies were first incubated in a dilution series for 30 min on ice and then labeled with anti-human IgG-PE. For quantitation, BD Quantibrite™ PE Phycoerythrin Fluorescence Quantitation Kit (BD Biosciences, lot #76536) was used. For the multi-color labeling experiment, fluorescent intensities were compensated using BD™ CompBeads Anti-Mouse Ig, κ/Negative Control Compensation Particles Set (BD Biosciences), and the performance of two anti-CD25 antibodies was confirmed to be equivalent. The cells were analyzed using a BD LSRFortessa Cell Analyzer (BD Biosciences) and data was analyzed using FlowJo_v10.8.1 (FlowJo, LLC).

### Epitope mapping

The epitopes of the five mAbs were determined using the multiple constructs listed in Supplementary Data 1. Vectors encoding human TNFR2, mouse TNFR2, and partially substituted mutants were constructed in an expression vector based on pcDNA3.1, which contains an internal ribosome entry site element to co-express TNFR2 with a reporter TagBFP. The expression vector was transfected into HEK293T cells using PEI’MAX’ (Polysciences Inc.). Cells were cultured for 40 h post-transfection and detached from the culture vessels using trypsin/EDTA for 5 min. Cells expressing wild-type and mutant TNFR2 were labeled covalently by combinations of succinimidyl ester compounds of Pacific Orange, DyLight 633, or DyLight 800 (Cat. No. P30253, 46414, and 46421; Thermo Fisher Scientific). The labeling agents were mixed in 12 different combinations of Pacific Orange (1 mg/mL), DyLight 633 (0.03 mg/mL), DyLight 800 (0.01 mg/mL or 0.3 mg/mL) or their absence, and the cells were incubated with the agents in PBS at pH 7.8 with 3% DMSO for 20 min at 31 °C. Twelve differently labeled cells were combined, mAbs (1.5 μg/mL) and the secondary antibody were incubated, and the cells were analyzed as described above.

### Reporter assay

TNFR2-expressing Ramos-Blue cells were seeded at 5 × 10^4^ cells/well in 100 μL of medium-containing antibodies at the indicated concentrations (100 fM–100 nM in tenfold dilution series) in the absence or presence of 50 ng/mL TNFα (Peprotech #AF-200-01A). The cells were incubated for 18 h, and secreted alkaline phosphatase was analyzed using p-nitrophenyl phosphate. Colorimetric changes were determined by measuring absorbance at 405 nm using EnSpire microplate reader (PerkinElmer, Waltham, MA, USA). Signals were normalized to the average absorbance of the eight wells incubated without antibody or TNFα (negative) and the eight wells incubated only with 50 ng/mL TNFα (positive). For the chart showing the agonistic activity, the negative and positive values were set at 0.1 and 0.9, respectively. For charts showing antagonistic activity, the values were set at −0.9 and −0.1, respectively.

### Size determination of immunocomplexes

Size-exclusion chromatography coupled with a multi-angle light scattering detector (SEC-MALS) was conducted, using a Superose 6 Increase 10/300 GL column (Cytiva) column and DAWN 6 (Wyatt, Santa Barbara, CA, USA) detector. PBS was used as the running buffer and the run was conducted at 0.5 mL/min. Under typical conditions, 2 μM antibody was mixed with 2 or 16 μM sTNFR2 and incubated for 10–20 min at room temperature. Data were analyzed using ASTRA 6 software (Wyatt). The protein concentration was calculated from the refractive index using d*n*/d*c* = 0.169. Molar mass values were determined by the Debye fitting of angle-dependent light scattering.

Mass photometry was conducted using Refeyn One (Refeyn Ltd., Oxford, UK). Five microliters of BpAbs (50 nM) mixed with sTNFR2 (50 or 400 nM) in PBS were diluted four-fold with 15 μL PBS pre-loaded for hydration on a glass slide. Interferometric scattering was observed under a microscope for 1–2 min, and the accumulated interference signal was analyzed using software provided by the manufacturer in the same way as literature^43^.

### Negatively stained electron microscopy

Bp109-92 and Bp45-94 in complex with sTNFR2 were prepared in a 1:1 mixture (1.3 μM) and stained without purification. A Bp109-92 F(ab′)_2_ complex with TNFR2 was prepared as a 1:4 mixture (2.6 μM of F(ab′)_2_ and 10.2 μM of TNFR2) at room temperature for 30 min, and purified in a Superose 6 Increase 10/300 GL column. The sample solutions were diluted to ~0.1 mg/mL and applied to carbon-coated copper grids which were glow-discharged for 20 s at 20 mA using an ion coater IB-3 (Eiko, Tokyo, Japan), and negatively stained with 2% (w/v) uranyl acetate. Micrographs were recorded at a nominal magnification of 25,000×, corresponding to 8 Å/pixel, using a JEM-1011 transmission electron microscope (JEOL, Tokyo, Japan), operating at 100 kV with a TemCamF416A-Hs-4 CMOS camera (TVIPS, Gauting, Germany). Image processing procedures, including particle selection, 2D classification, and averaging were performed using the RELION program^44^.

### Cryo-EM specimen preparation and data collection

The Bp109-92 and TNFR2-MBP complex was prepared as a 2:3 mixture (5.6 μM Bp109-92 and 8.4 μM TNFR2-MBP) at room temperature for 5 min, and purified in a Superose 6 Increase 10/300 GL column. Three microliters of the complex solution (0.2 mg/mL) were applied to a Quantifoil R1.2/1.3 Cu 200 mesh grid (Quantifoil Micro Tools GmbH, Jena, Germany) that was glow-discharged for 20 s at 20 mA using a JEC-3000FC sputter coater (JEOL). The grid was blotted with a blot force of 0 and a blot time of 3 s in a Vitrobot Mark IV chamber (Thermo Fisher Scientific) equilibrated at 4 °C and 100% humidity, and then immediately plunged into liquid ethane. Excess ethane was removed with filter paper, and the grids were stored in liquid nitrogen. The image dataset was collected using SerialEM^45^, yoneoLocr^46^, and JEM-Z300FSC (CRYO ARM 300: JEOL) operated at 300 kV with a K3 direct electron detector (Gatan, Pleasanton, CA, USA) in CDS mode. The Ω-type in-column energy filter was operated with a slit width of 20 eV for zero-loss imaging. The nominal magnification was 60,000×. Defocus varied between −0.5 and −2.0 μm. Each movie was fractionated into 60 frames (0.081 s each, total exposure: 4.87 s), with a total dose of 60 e^−^/Å^2^.

### Cryo-EM image processing and model building

A gain reference image was prepared with the relion_estimate_gain command in RELION 4.0^44^ using the first 500 movies. Images were processed using cryoSPARC ver. 3.3.2^47^. A total of 5724 movies were imported and motion corrected, and contrast transfer functions (CTFs) were estimated. A total of 4160 micrographs with maximum CTF resolutions greater than 5 Å were selected. First, the particles were automatically picked using a blob picker job with a particle diameter between 100 and 150 Å. After particle extraction with 4× binning, 2D classification into 50 classes was performed to select clear 2D class averages as templates for subsequent particle picking. Then, the particles were again automatically picked with the templates, and 2,218,071 particle images were extracted with a box size of 320 pixels using 4× binning (downscaled to 80 pixels). Two rounds of 2D classification into 50 classes, with a circular mask of 170 Å, were performed to select 354,477 particles. The number of final full iterations and the batch size per class were increased to 20 and 200, respectively. The best initial model was selected from four reconstructed models. A total of 100,391 particles belonging to the best model were extracted again with a box size of 320 pixels without binning. After homogeneous refinement, global/local CTF refinement and nonuniform refinement^48^ were performed to reach 4.26 Å overall map resolution. The particle images were downsampled to 256 pixels (corresponding to a pixel size of 1.088 Å). After one round of nonuniform refinement, the handedness of the map and mask was flipped. After another round of nonuniform refinement and two rounds of local CTF refinement and nonuniform refinement, a final map was reconstructed at 3.63 Å resolution (FSC = 0.143).

Homology models of 109-Fab and 92-Fab were generated using SWISS-MODEL^49^. The atomic model of TNFR2 (PDB entry:3ALQ) and the homology models of Fabs were manually fitted into the density and modified using UCSF Chimera^50^ and Coot^51^. Realspace refinement was performed using the PHENIX software^52^. Several rounds of manual model modification and real space refinement were performed. Figures were prepared using UCSF Chimera^50^ and PyMOL (Schrödinger, LLC). The parameters are summarized in Table 1.

### Stimulation of PBMCs

PBMCs were passed through a 40 μm cell strainer and diluted to 1 × 10^5^ cells/well in a 96-well round-bottom plate and were cultured in the presence of 10 ng/mL IL-2 (#Z00368; GenScript Biotech, Piscataway, NJ, USA), the presence or absence of TNFα (50 ng/mL), and antagonists (50, 150, and 500 ng/mL). The cells were incubated at 37 °C for 48 h. Following incubation, cells were labeled using the LIVE/DEAD™ Fixable Near IR (780) Viability Kit (Thermo Fisher Scientific) and analyzed using flow cytometry. eBioscience Intracellular Fixation & Permeabilization Buffer Set (Thermo Fisher Scientific) was used to stain Foxp3. For cluster analysis, the whole PBMC population, in eight conditions (10000 counts per condition; Fig. 5a, b), were clustered using five parameters (CD3-BV711, CD4-BV510, CD25-BV421, CD127-PerCP-Cy5.5, Foxp3-AlexaFluor 647). One thousand steps of iteration by fast Fourier transform-accelerated interpolation-based t-distributed stochastic neighbor embedding (FIt-SNE) implemented in FlowJo v10.8.1, was conducted^53^. CD25-BV605 was used instead of CD25-BV421 for comparative evaluation of anti-TNFR2 F(ab′)_2_ with TR109 or Bp109-92.

### Statistics and reproducibility

The number of replicates is shown in each figure legend. For biological experiments, replicates are defined as the replicated experiments conducted using independently prepared materials (diluted into the working buffer or medium). At least three experiments were conducted except for a binding experiment shown in Fig. S7. Tukey’s honest significance test was conducted using R 4.3.0 for testing populations by PBMC stimulation. Error bars represent standard deviation except for Fig. S7, shown with standard error.

### Reporting summary

Further information on research design is available in the Nature Portfolio Reporting Summary linked to this article.

### Supplementary information

Supplementary Information

Description of Additional Supplementary Files

Supplementary Data 1

Supplementary Data 2

Reporting Summary

## Data Availability

Cryo-EM density map and model of Bp109-92 in complex with TNFR2-MBP are deposited to Electron microscopy Data Bank and Protein Data Bank with accession codes EMD-34871 and PDB-8HLB, respectively. Sequences of the antibody variable regions used are described in the related patent (WO2021200840). Source data for Fig. 2e, Fig. 3e, f, and Fig. 5a, b are available as Supplementary Data 2. Other data and materials are available from the corresponding authors upon reasonable request.
