# Supplementary material for: Development of a 1:1-binding biparatopic anti-TNFR2 antagonist by reducing signaling activity through epitope selection
Source: Commun Biol. 2023 Sep 27;6:987. doi: 10.1038/s42003-023-05326-8 (PMC10533564; doi:10.1038/s42003-023-05326-8)
Supplement: Supplementary file 1 — Supplementary Information [file 42003_2023_5326_MOESM1_ESM.pdf]

# Supplementary Information for

## Development of a 1:1-binding biparatopic anti-TNFR2 antagonist by reducing signaling activity through epitope selection

Hiroki Akiba[1,2]*, Junso Fujita[3,4,5], Tomoko Ise[2], Kentaro Nishiyama[1], Tomoko Miyata[3,4], Takayuki Kato[6], Keiichi Namba[3,4,7], Hiroaki Ohno[1,2], Haruhiko Kamada[1,2], Satoshi Nagata[2]*, and Kouhei Tsumoto[2,8,9]*

[1] Graduate School of Pharmaceutical Sciences, Kyoto University; Sakyo-ku, Kyoto, 606-8501, Japan.
[2] Center for Drug Design Research, National Institutes of Biomedical Innovation, Health and Nutrition; Ibaraki City, Osaka, 562-0011, Japan.
[3] Graduate School of Frontier Biosciences, Osaka University; Suita City, Osaka, 565-0871, Japan.
[4] JEOL YOKOGUSHI Research Alliance Laboratories, Osaka University; Osaka, 565-0871, Japan.
[5] Graduate School of Pharmaceutical Sciences, Osaka University; Suita City, Osaka, 565-0871, Japan.
[6] Institute of Protein Research, Osaka University; Suita City, Osaka, 565-0871, Japan.
[7] RIKEN SPring-8 Center; Osaka 565-0871, Japan
[8] School of Engineering, The University of Tokyo; Bunkyo-ku, Tokyo, 113-8656, Japan.
[9] Institute of Medical Sciences, The University of Tokyo; Minato-ku, Tokyo, 108-8639, Japan.

*Corresponding authors.

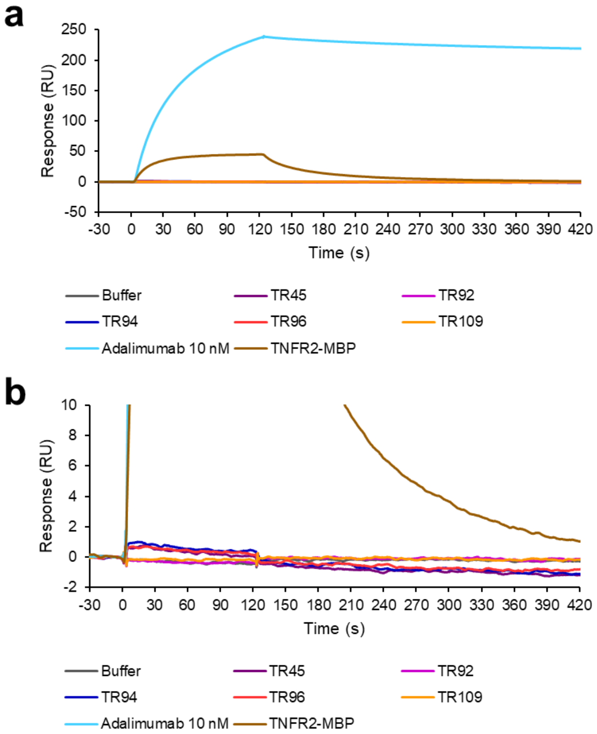


**Fig. S1. Interaction of the five antibodies with TNFα measured by surface plasmon resonance.** To the immobilized TNFα (120 RU) on a sensor chip, 100 nM anti-TNFR2 antibodies, 10 nM adalimumab (anti-TNFα) or 100 nM TNFR2-MBP was flowed. **a**, Sensorgrams showing the interaction of antibodies or TNFR2-MBP to TNFα. **b**, Enlarged view from **a**.

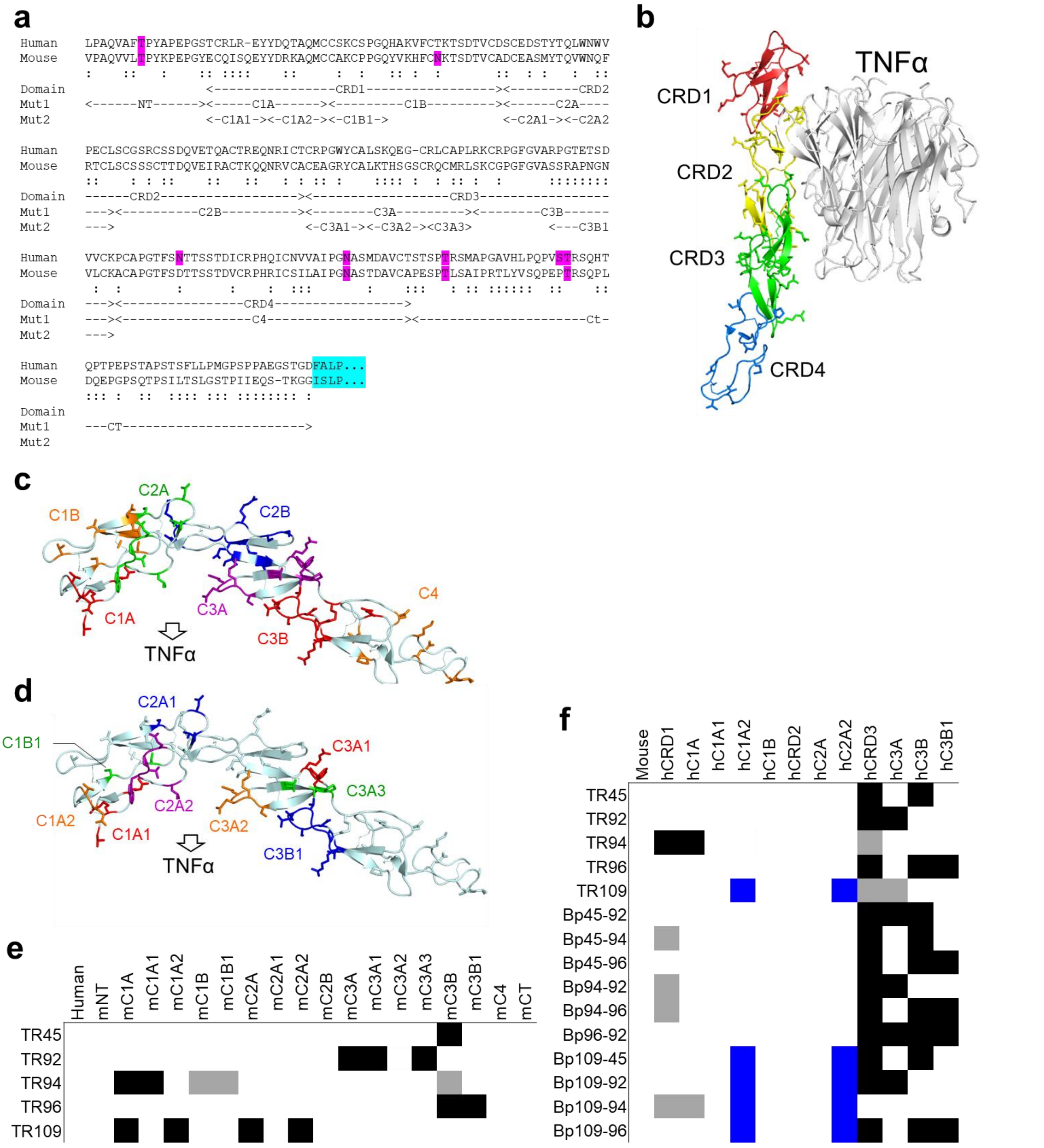


**Fig. S2. a-d, Design of mutants for determining the antibody epitopes. a,** Alignment of the extracellular regions of human TNFR2 (UniProt ID: P20333) and mouse TNFR2 (UniProt ID: P25119) and the analyzed mutants. The residues colored purple are the potential glycosylation sites and the residues colored cyan are the N-termini of transmembrane helix. ':' indicates different amino acids between the two orthologs. Human-to-mouse mutants were designed so that the peptide sequences of the indicated regions on Mut1 or Mut2 lines were replaced with that of mouse TNFR2. Mouse-to-human mutants were designed in the opposite manner. **b,** Domains mapped onto the tertiary structure of human TNFR2 in complex with TNFα (PDB entry: 3ALQ). One TNFR2 molecule and trimeric TNFα are shown. **c,d,** Mutated amino acids of each mutant in the Mut1 (**c**) and Mut2 (**d**) series are labeled on the structure of TNFR2. In **b**–**d**, CT and NT regions are disordered and not shown. **e,f,** Simplified map of epitope regions determined by binding reduction for mouse-to-human mutants (**e**) or by increased binding for human-to-mouse mutants (**f**). For **e**, the regions were determined by reduced binding of the conventional IgGs to the cells expressing the mutants compared to human TNFR2. Black indicates loss of binding; grey indicates partially reduced binding. Reduction

is analyzed by flow cytometry presented in Fig. S3. For **f**, the regions were determined by increased binding of the conventional IgGs or BpAbs to the cells expressing the mutants compared to mouse TNFR2. Black indicates gain of binding; grey indicates partially gained binding; blue indicates gain of binding when two regions were mutated at the same time. Reduction is analyzed by flow cytometry presented in Fig. S4.

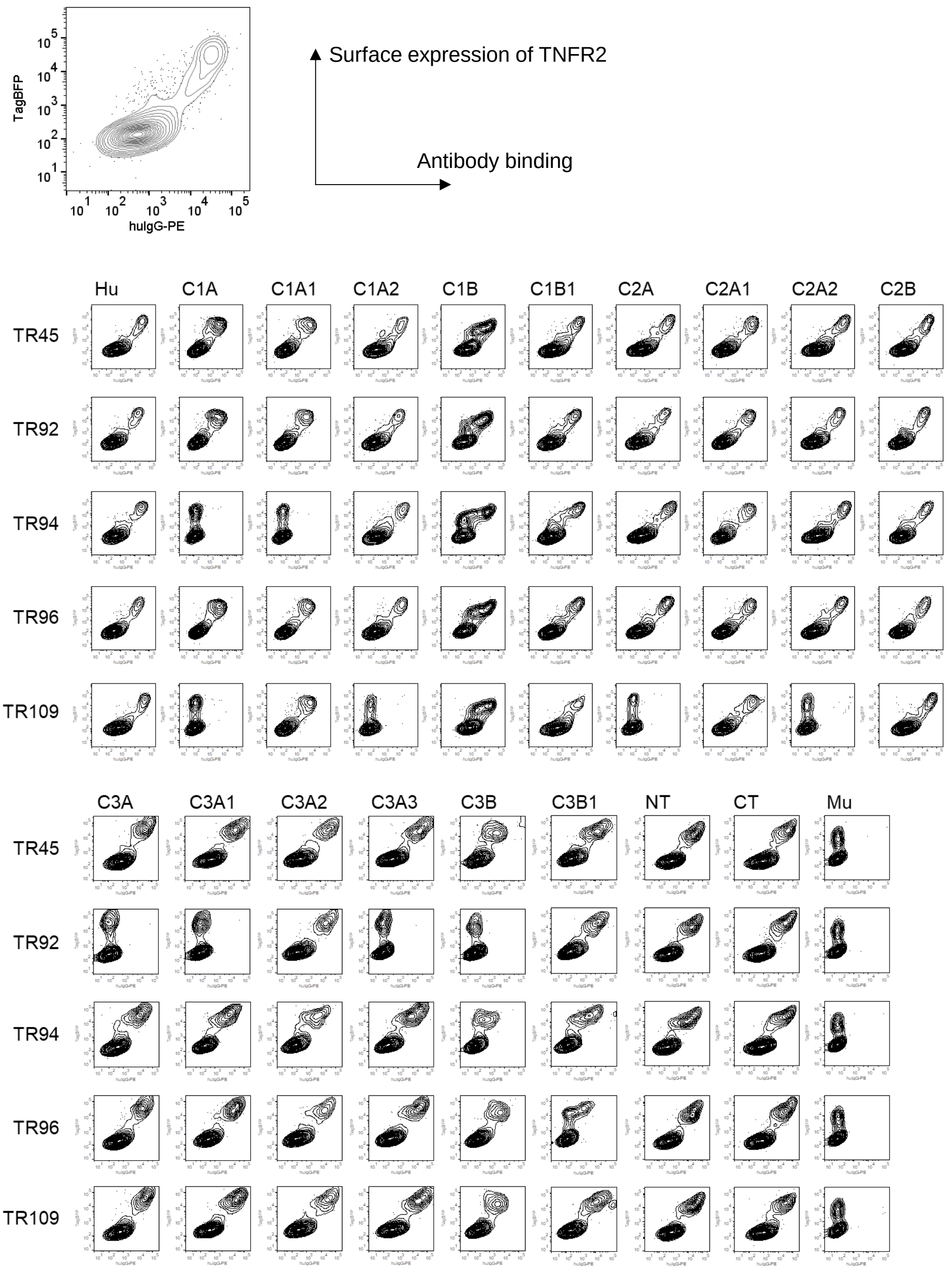


**Fig. S3. Binding of monoclonal antibodies to the wild-type and mutant human TNFR2 series.** Expression of TNFR2 was visualized by TagBFP as a bicistronic reporter (y-axis). Binding of the antibody was visualized by secondary antibody conjugated with R-PE (x-axis). Reduced antibody binding to wild-type or human-to-mouse mutant TNFR2$^+$ cells indicates that the mutated region is the epitope of the antibody.

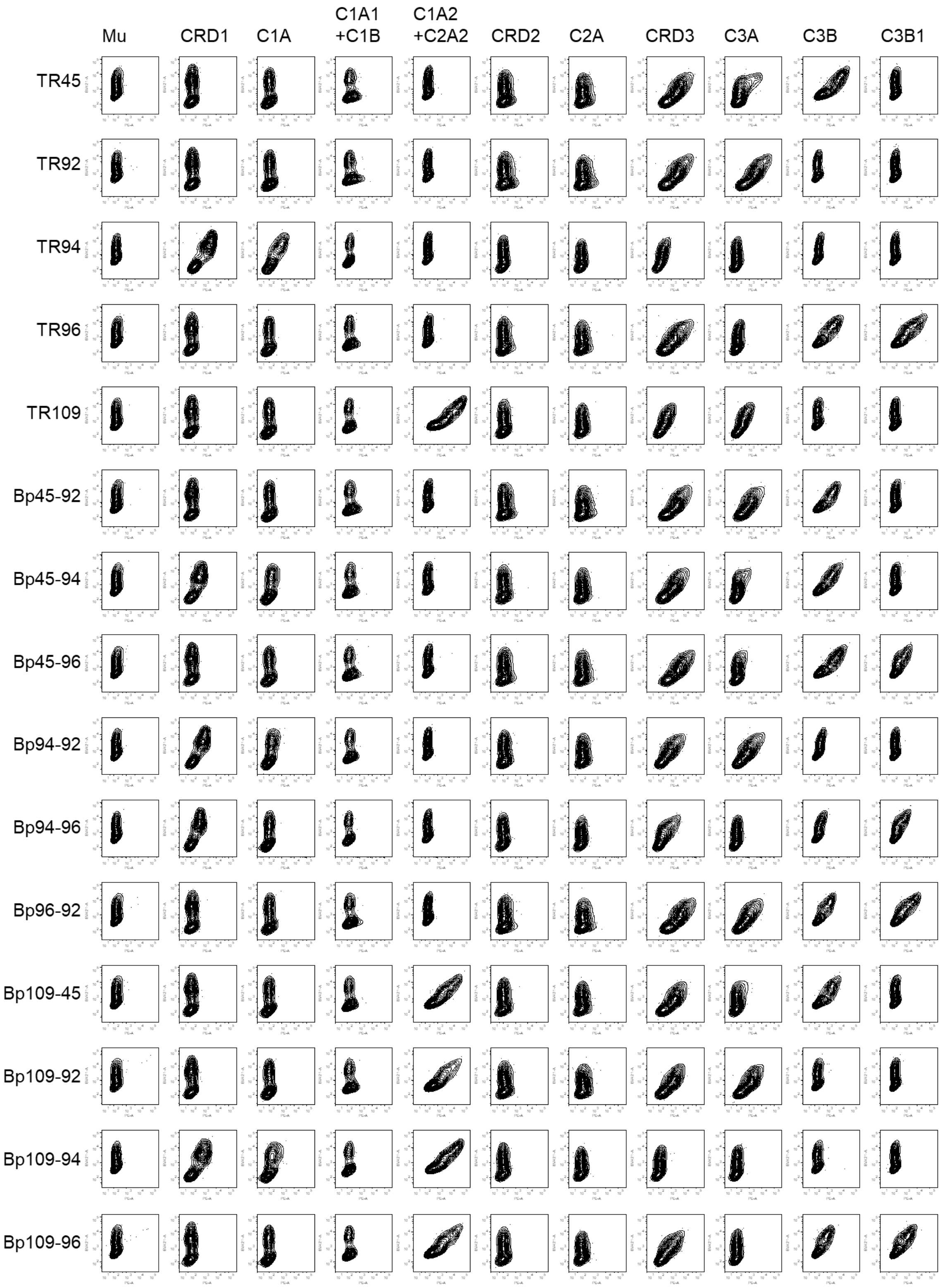


**Fig. S4. Binding of chimeric monoclonal antibodies and biparatopic antibodies to the wild-type and mutant murine TNFR2 series.** Expression of TNFR2 was visualized by TagBFP as a bicistronic reporter (y-axis). Binding of the antibody was visualized by secondary antibody conjugated with R-PE (x-axis). Antibody binding to mouse-to-human mutant TNFR2$^{+}$ cells indicates that the mutated region is the epitope of the antibody.

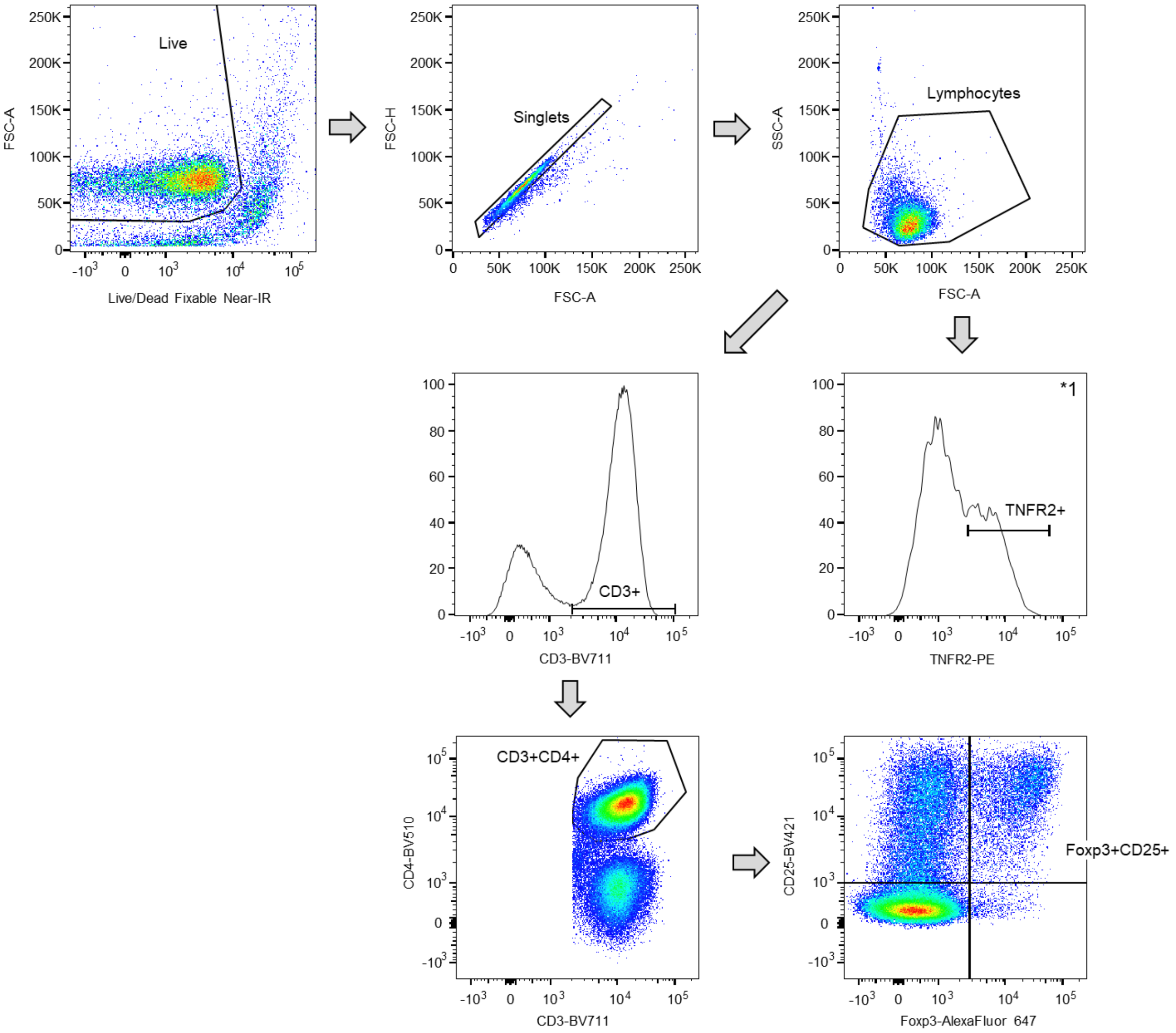


**Fig. S5. Representative gating strategy for analyzing peripheral blood mononuclear cells.** TNFR2 expression in lymphocytes in the presence of 50 ng/mL TNFα was used for gating for clarity and the same gating was used for all conditions (*1).

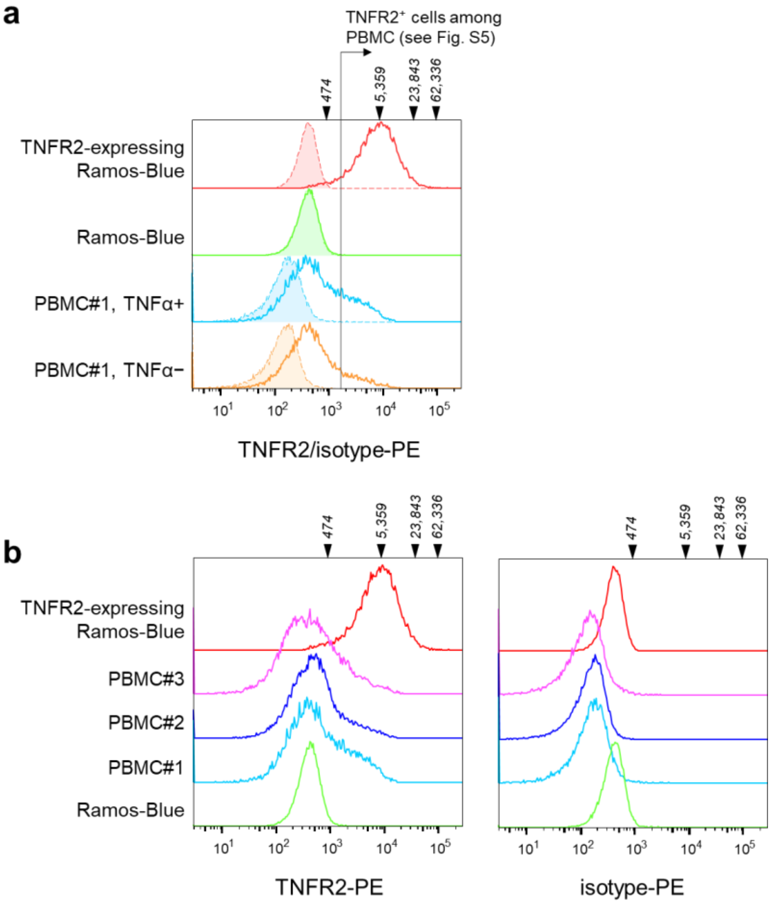


**Fig. S6. Comparison of TNFR2 expression level of TNFR2-expressing Ramos-Blue cells with peripheral blood mononuclear cells (PBMCs). a,** TNFR2 expression of the original Ramos-Blue cell line (green) and the TNFR2 transfectant (red) was compared with their TNFR2 expression of PBMCs cultured in the presence (TNFα+, cyan) or absence (TNFα−, orange) of 50 ng/mL of TNFα. Solid line, anti-TNFR2; shaded dashed line, mouse IgG2a isotype control. Gating strategy for 'lymphocytes' was conducted for the PBMCs as shown in Fig. S5. PBMCs contain various cells expressing different level of TNFR2 either in the presence or absence of TNFα. A vertical line corresponding to $TNFR2^{+}$ gating threshold used in Fig. S5 is shown. Numerical values on top of the panel represents the number of R-PE corresponding to the fluorescence intensity determined by quantitation beads (beads of 474, 5359, 23843, and 62236 PE molecules per bead showed mean fluorescence intensity of 859, 8247, 35255, and 94666, respectively). The maximum TNFR2 expression level of TNFα-stimulated PBMC was ~5,000 (fluorescence intensity of ~8,000) per cell, which was almost identical to the mean value of TNFR2-expressing Ramos-Blue (4,400, calculated from the mean fluorescence intensity of 7,100). **b,** Comparison of three different lots (cyan, blue and purple) of PBMC stimulated with 50 ng/mL TNFα. Left panel, anti-TNFR2; right panel, isotype control.

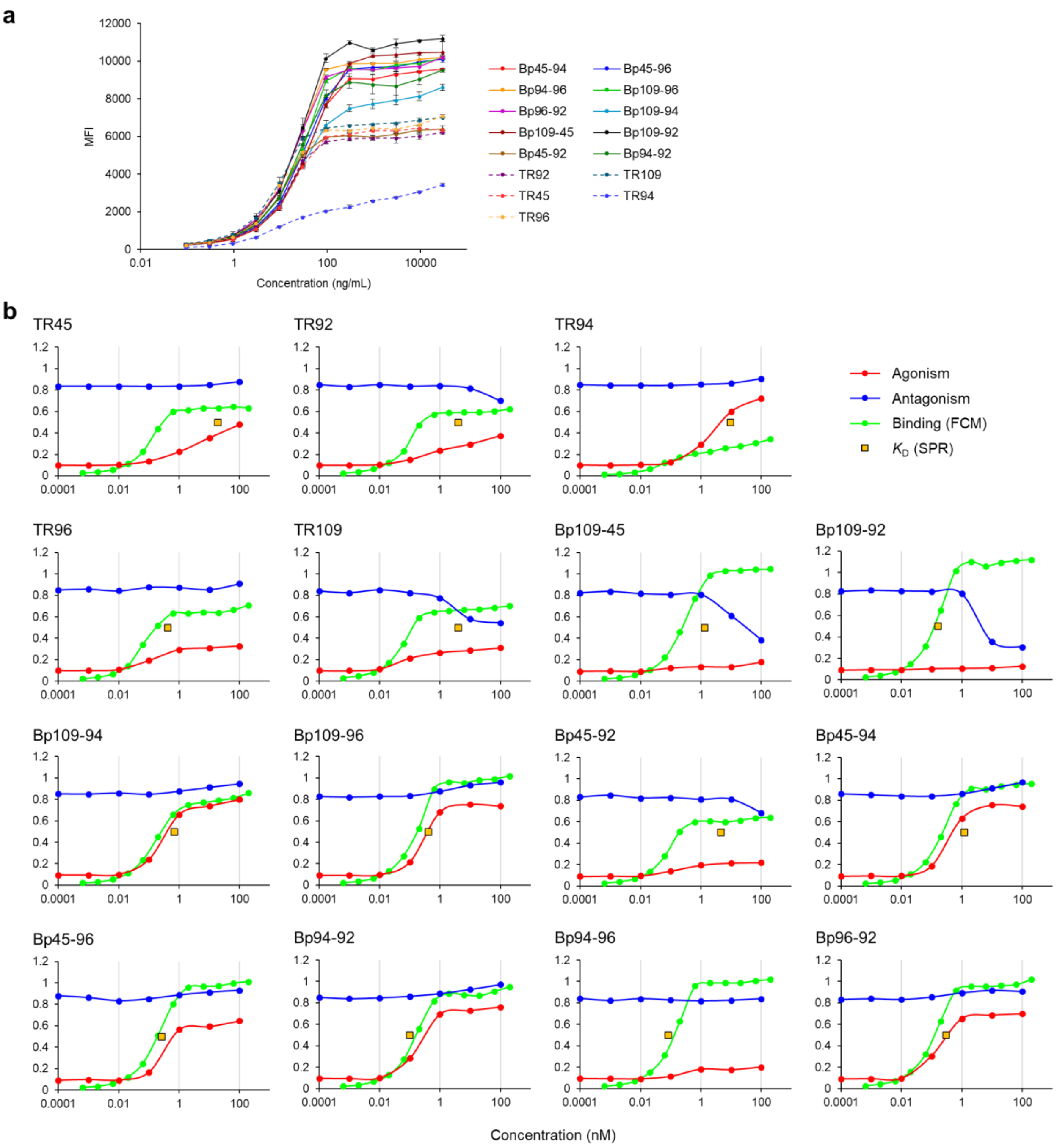


**Fig. S7. Binding activities of the antibodies and comparative analysis with biological activities. a,** Concentration-dependent binding of antibodies to TNFR2-expressing Ramos-Blue cells. Binding was detected using anti-human IgG R-PE, and mean fluorescence intensity (MFI) was obtained in two independent experiments. To standardize the experiments, the MFI values for three highest concentrations were averaged for each antibody (= $A_i$; i, each antibody). $\Sigma(kA_i^{1st} - A_i^{2nd})$ was minimized for $A$ values of ten antibodies, excluding those with TR94 variable regions as they did not reach a plateau. Using the standardized MFI, the average values are shown with standard error. **b**, Comparative analysis. For all panels, x-axes are concentration (nM) and y-axes are arbitrary units shared for all 15 antibodies. Data are adopted from Fig. 2e and Table S1. Red, biological activity (upregulation by agonistic activity); blue, biological activity in the presence of TNFα (downregulation by antagonistic activity); green, binding activity by flow cytometry; yellow square, dissociation constant against TNFR2.

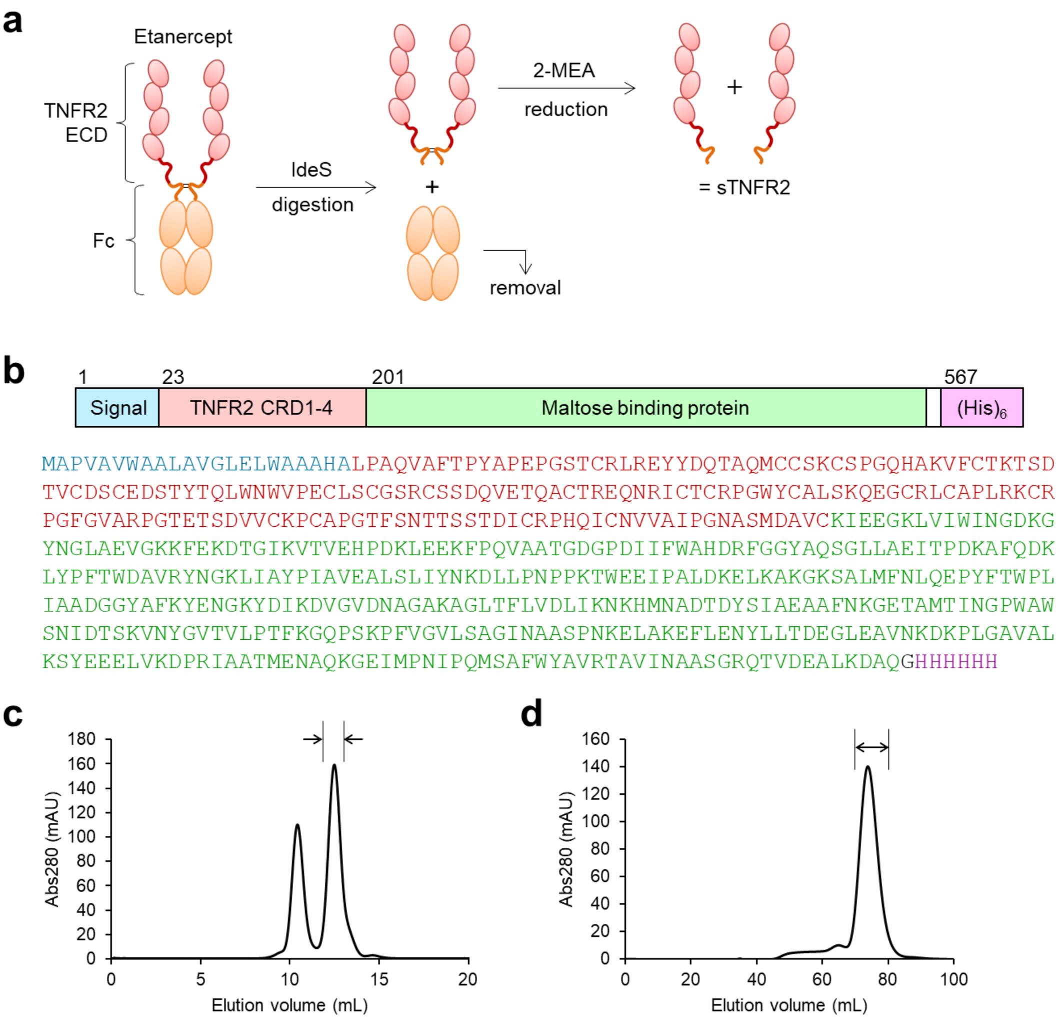


**Fig. S8. Recombinant TNFR2 proteins used for physicochemical and structural analyses. a.** sTNFR2 obtained from etanercept. **b.** TNFR2-MBP construct. **c.** Size-exclusion chromatogram of sTNFR2 using Superdex200 increase 10/300 column. **d.** Size-exclusion chromatogram of TNFR2-MBP using Superdex200 16/600 column. The arrows indicate the collected fractions.

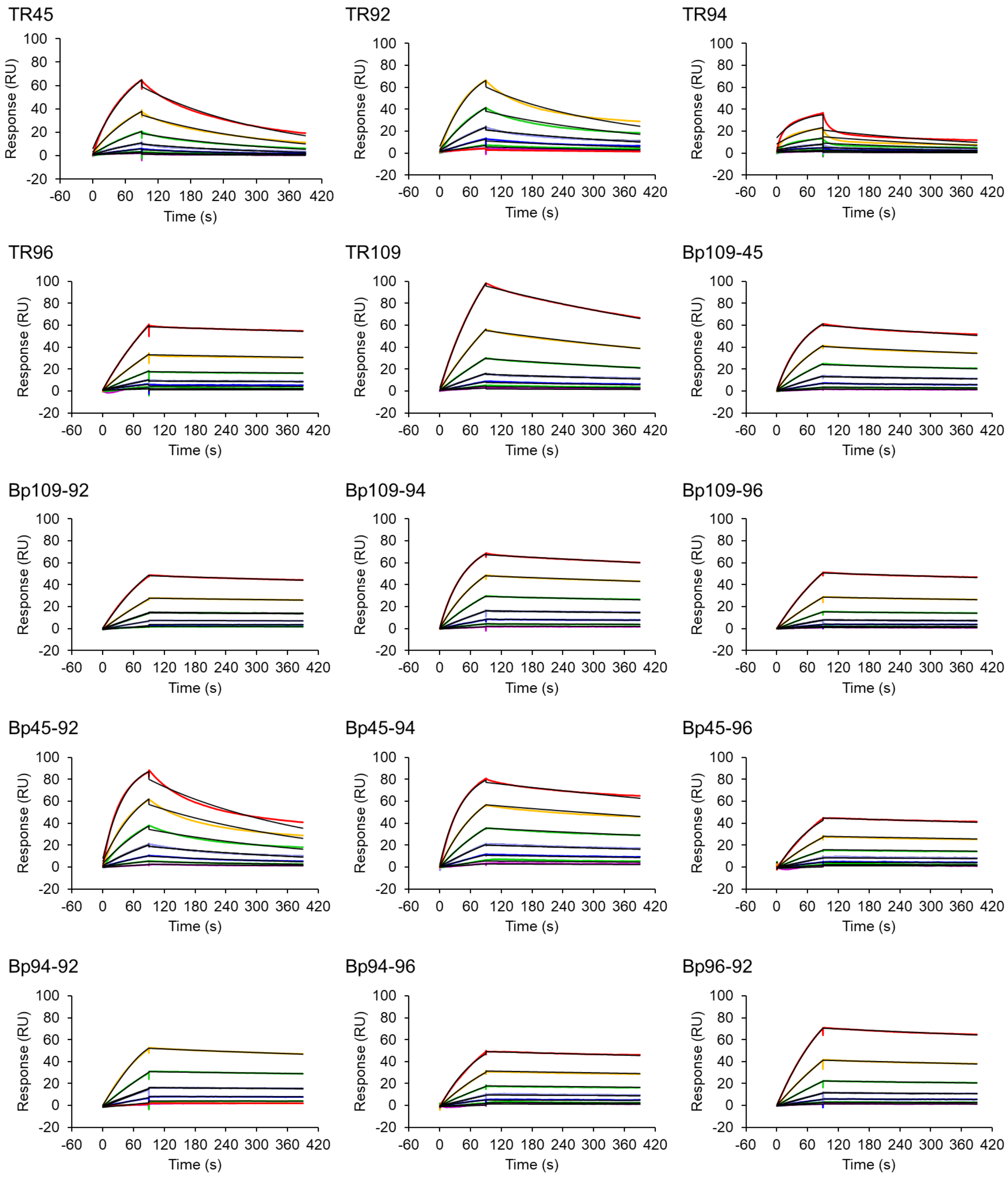


**Fig. S9. Surface plasmon resonance sensorgrams of the immobilized antibody binding to sTNFR2.** sTNFR2 as the analyte was used in a two-fold dilution series of different concentration ranges dependent on the immobilized antibody. The concentration was 1.25 – 80 nM (TR94 cIgG), 0.156 – 10 nM (cIgG and BpAbs bearing TR96 variable region), 0.313 – 10 nM (Bp94-92 and Bp109-92), 0.625 – 20 nM (TR92 cIgG), or 0.625 – 40 nM (others). See Table S1 for the kinetic parameters. Binding of TR94 was not strong and the parameters were determined poorly by fitting to 1:1 binding kinetics.

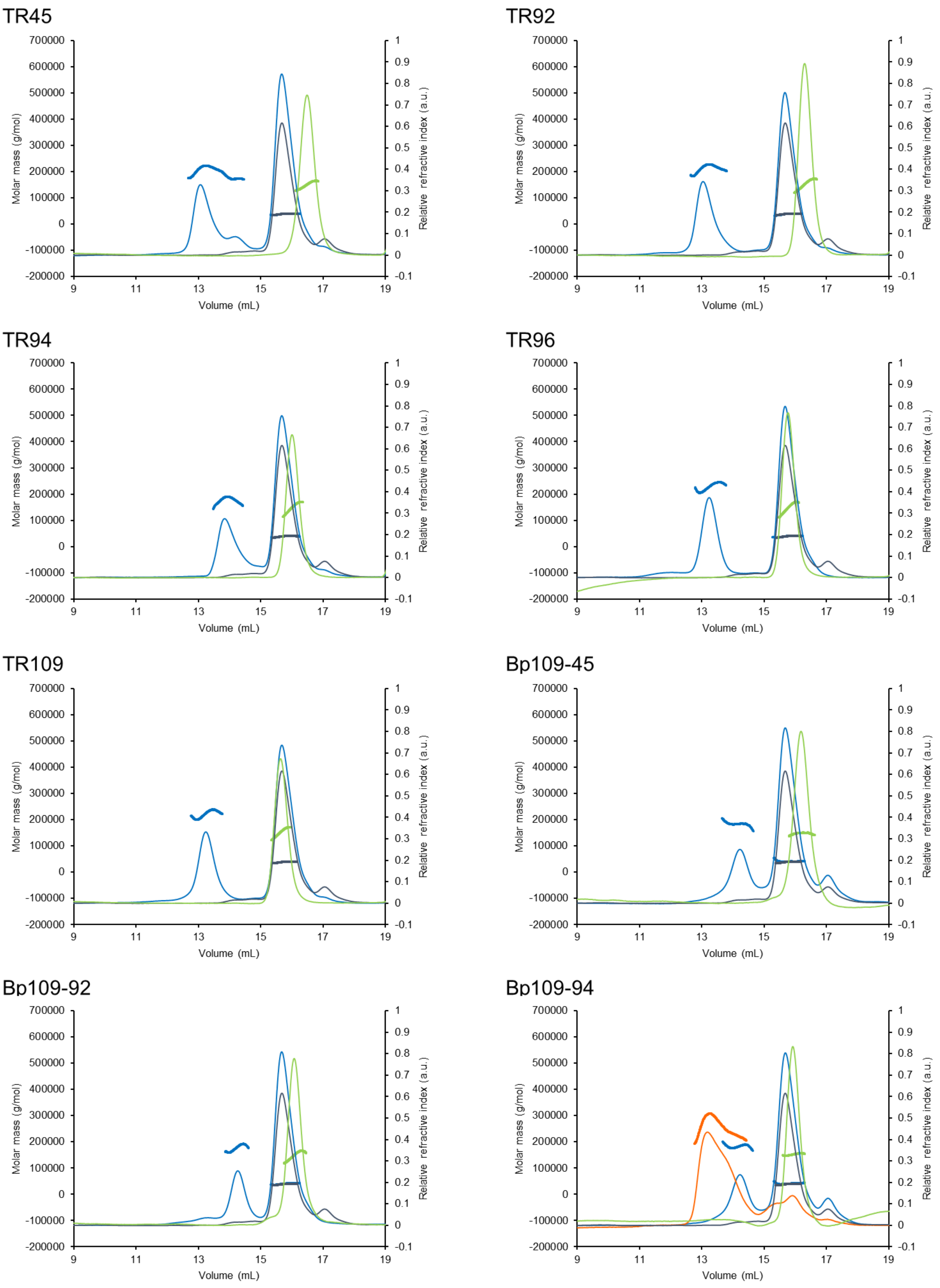


**Fig. S10.** *Continued to next page.*

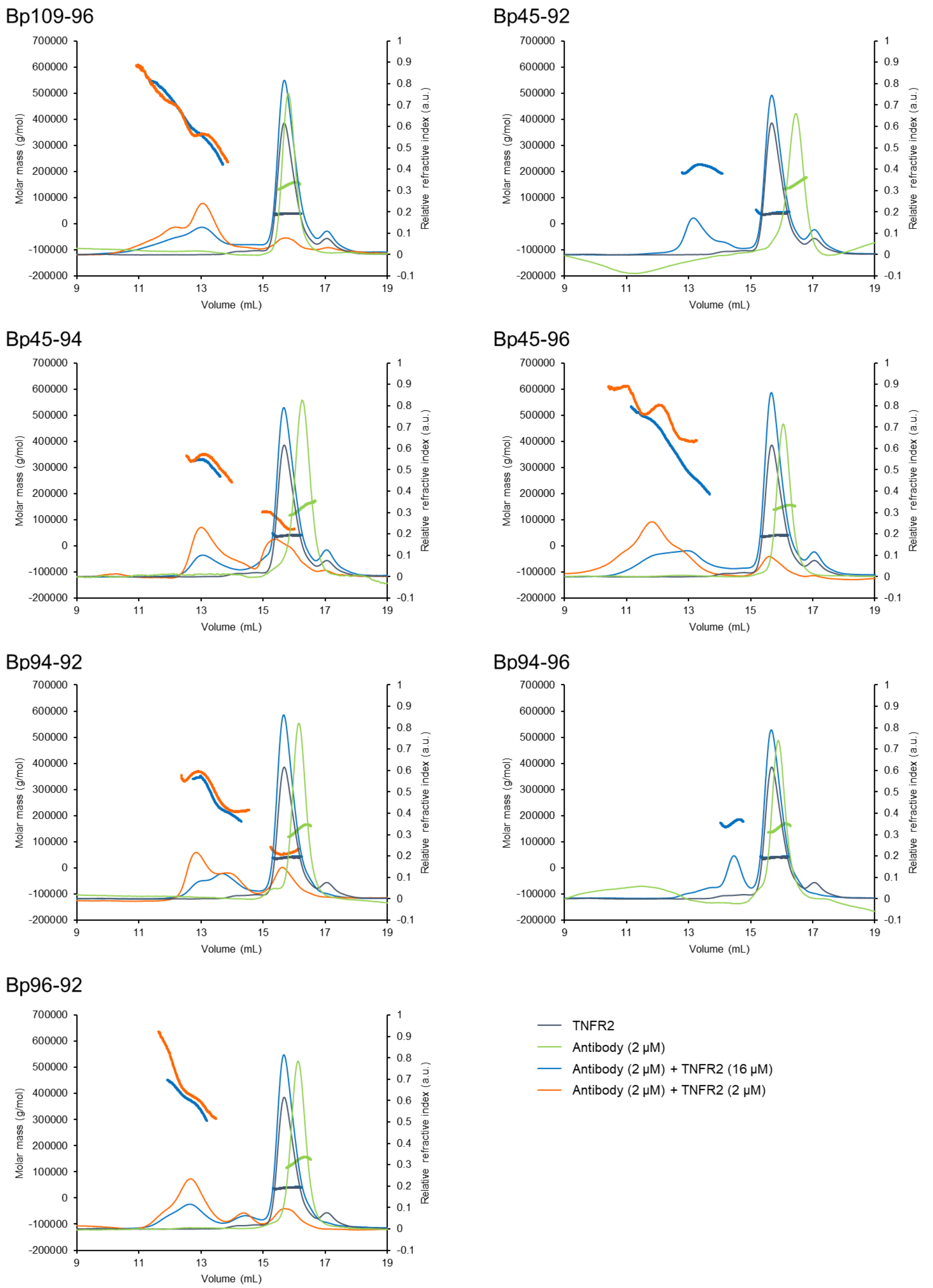


**Fig. S10. SEC-MALS charts.** For each chromatogram, relative refractive index is shown with thin lines (right y-axis), and the molar mass is shown with bold lines (left y-axis).

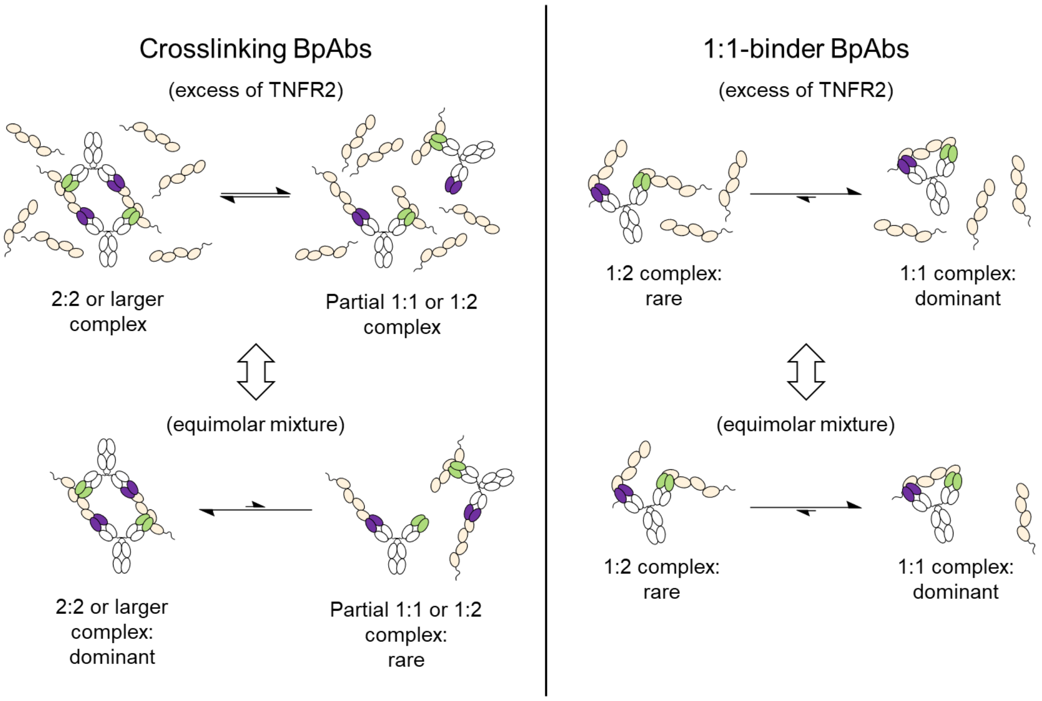


**Fig. S11. Proposed equilibrium of complex formation in solution using recombinant TNFR2.** In the case of bridging BpAbs (left), large complexes and partial 1:1 or 1:2 complexes are in equilibrium in the presence of excess TNFR2. On the other hand, a large complex is dominant for equimolar mixture due to the absence of sufficient amounts of TNFR2 for 1:2 complex formation (Fig. 3e,f). In case of 1:1-binder BpAbs, 1:1 complex is dominant irrespective of the BpAb:TNFR2 ratio (Fig. 3d).

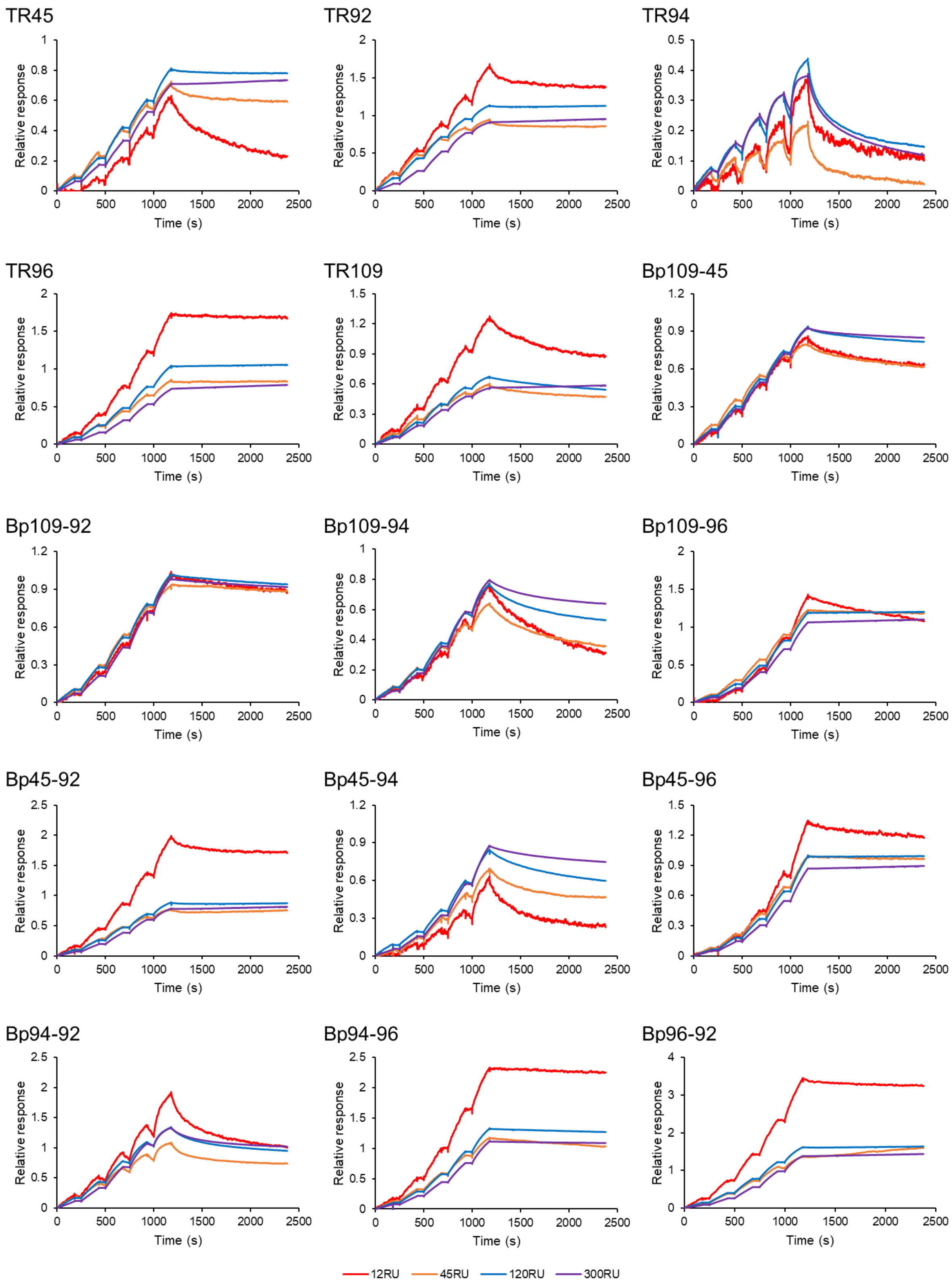


**Fig. S12. Surface plasmon resonance sensorgrams of the antibodies binding to immobilized TNFR2 in single-cycle kinetics.** TNFR2-MBP was captured by anti-MBP antibody to the level of 12 RU (red), 45 RU (orange), 120 RU (blue) or 300 RU (purple) and the antibodies (0.4 – 6.4 nM by twofold dilution series) were flowed over TNFR2-MBP. Fitted kinetic values are presented in Table S2. See Fig. S13 for raw sensorgrams.

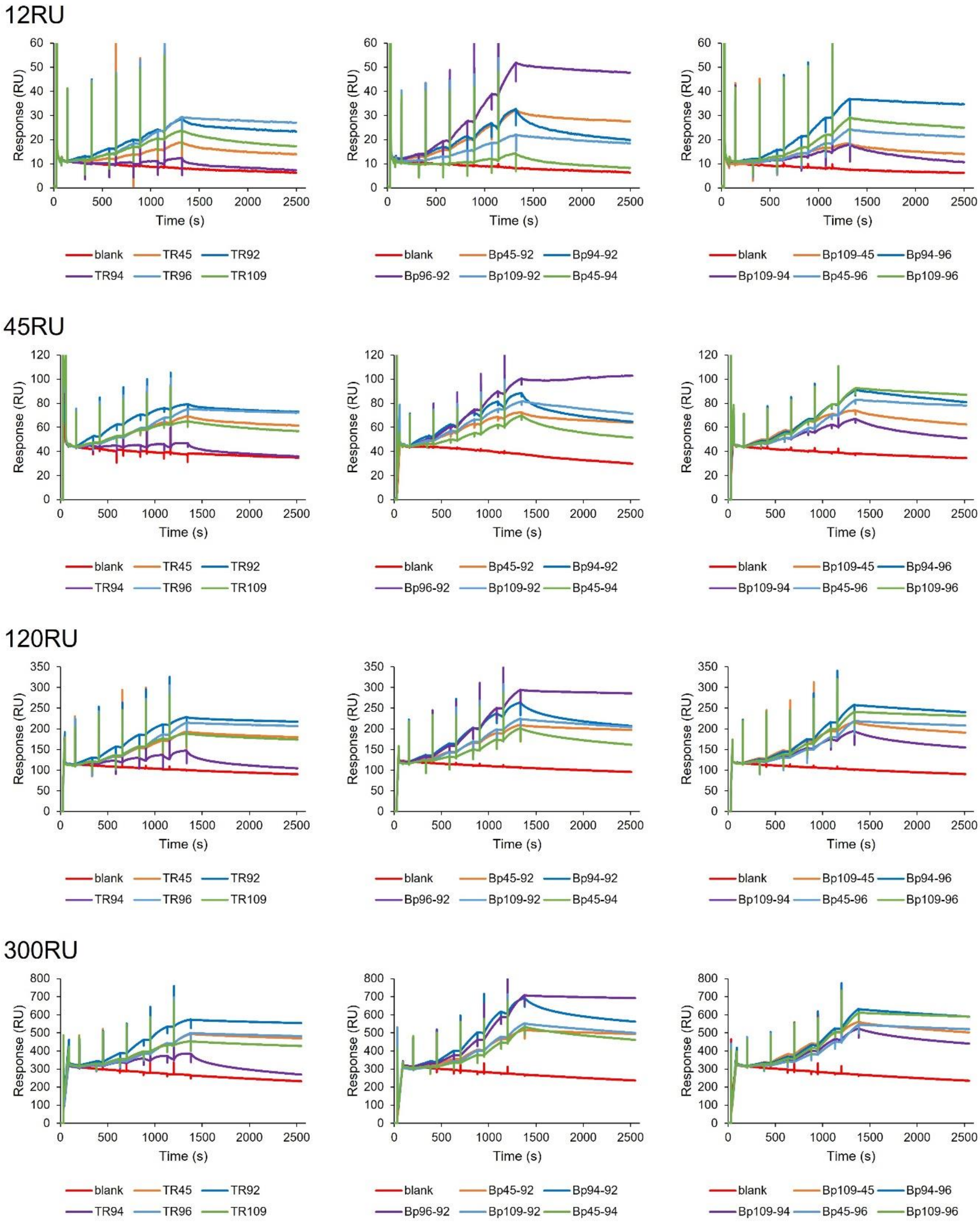


**Fig. S13. Raw sensorgrams for Fig. S12 including the capture step of TNFR2-MBP.** Baseline before capturing TNFR2-MBP is standardized as zero response. Regeneration steps are omitted. For presentation of Fig. S12, each sensorgram of antibody binding was subtracted by the blank run (in the absence of flowed antibody), and the response values were divided by the experimentally determined response values of captured TNFR2-MBP by each run (Table S2, 'Capture' column) to produce relative response values.

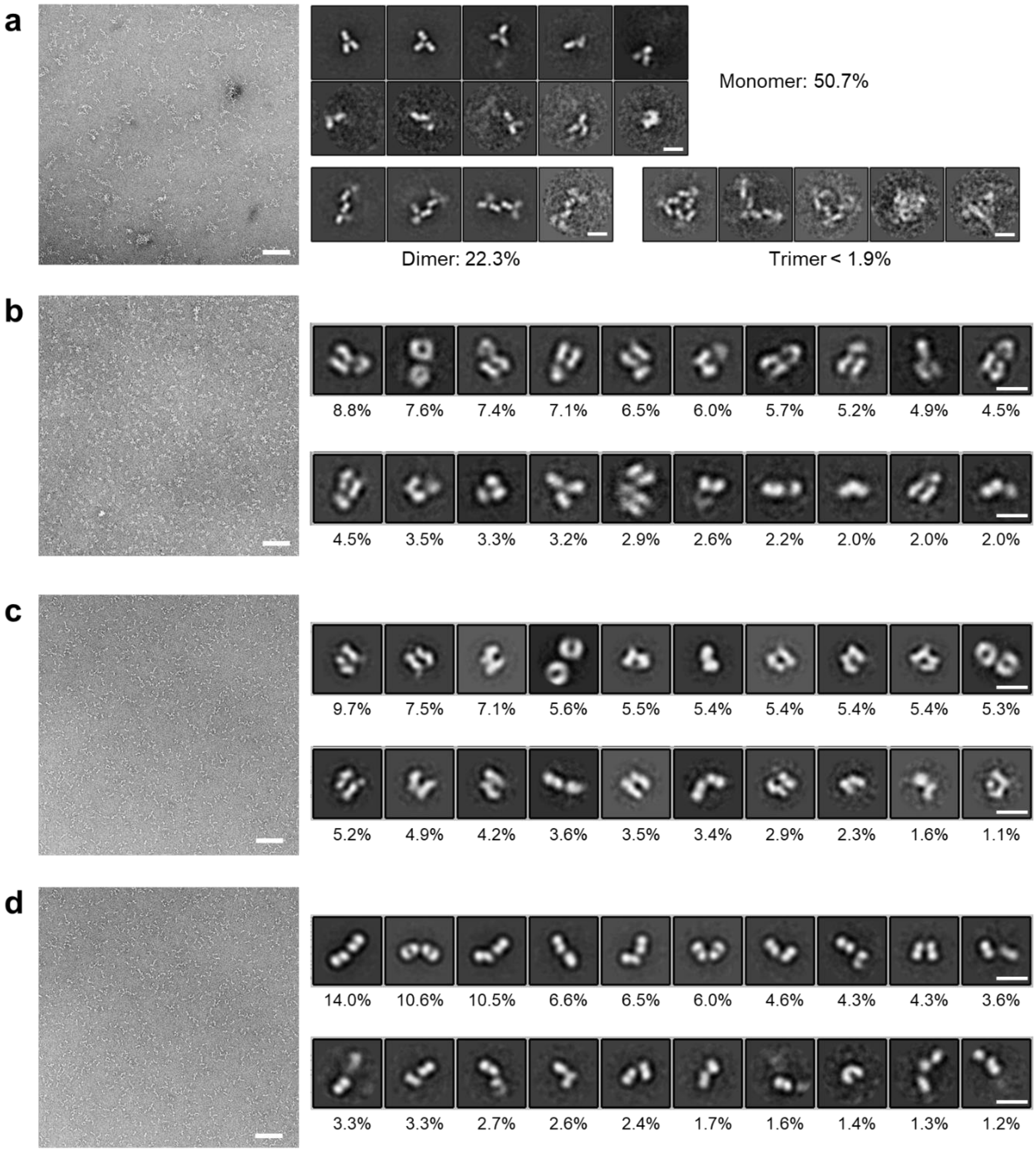


**Fig. S14. Negatively stained electron microscopic images of selected biparatopic antibodies in the presence (a-c) or absence (d) of TNFR2.** a, Bp45-92; b, Bp109-92; c,d, Bp109-92 F(ab′)$_2$. Left, representative images (Scale bar: 500 Å); right, particles classified and the percentage of each class of structures in 6,725 (a), 9,786 (b), 12,267 (c) and 13,046 (d) particles (Scale bar: 100 Å). For a, classes are defined by the number of IgG-like structural elements per particle. For b-d, top 20 classes are shown.

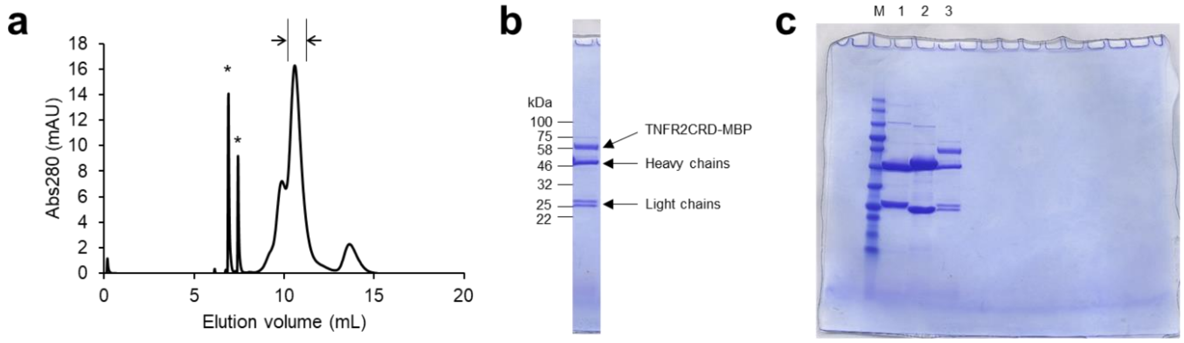


**Fig. S15. Preparation of samples for cryo-electron microscopy. a**, Size-exclusion chromatogram of Bp109-92–TNFR2-MBP complex using Superose 6 Increase 10/300 column. The arrows indicate the collected fractions. Asterisks are unrelated signals due to accidental stop of the fraction collector. **b**, CBB-stained SDS-PAGE of the obtained complex. **c**, Uncropped gel image for b. Lanes, M, size marker (Blue Prestain Protein Standard, Broad Range (11-190 kDa); NEB #P7706S); 1,2, unrelated proteins; 3, Bp109-92–TNFR2-MBP complex.

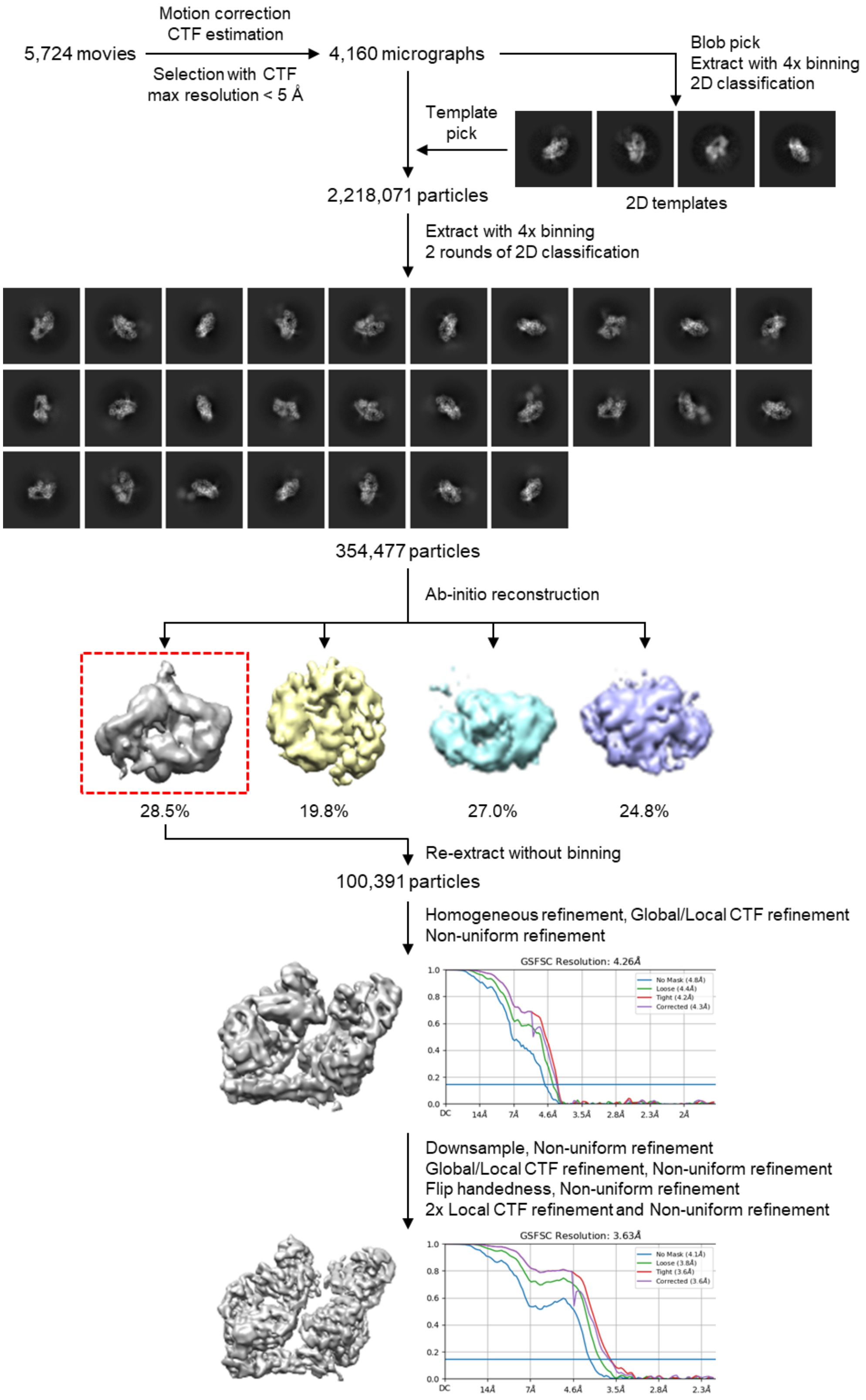


**Fig. S16. Image processing of Bp109-92–TNFR2-MBP complex.**

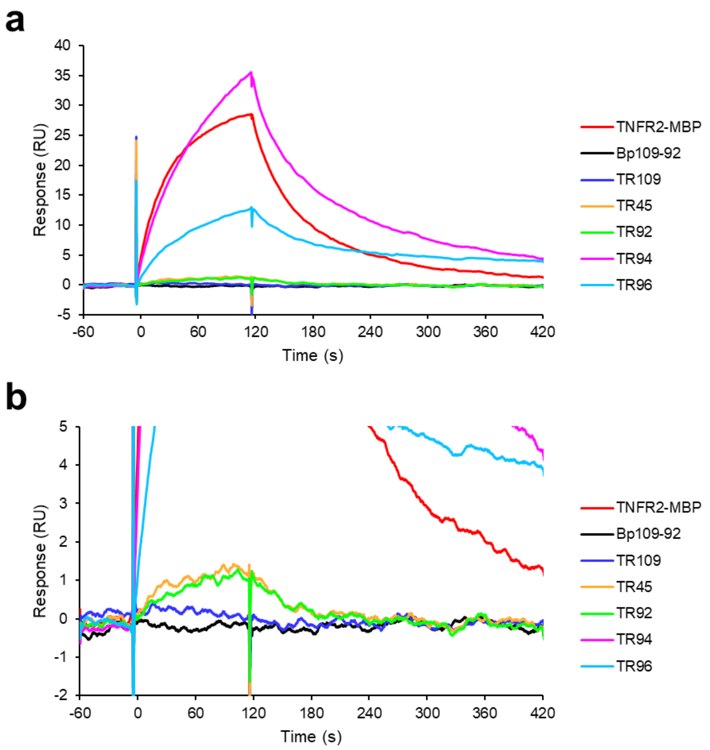


**Fig. S17. Ligand-blocking activity of the antagonist, Bp109-92, and five cIgGs analyzed by surface plasmon resonance.** To immobilized TNFα on a sensor chip, TNFR2-MBP (25 nM) or its mixture with twofold excess antibody was flowed. Bp109-92 and TR109 showed complete ligand-blocking activities, while TR92 and TR45 showed partial ligand-blocking activities. Although mutant-defined TR45 epitope is not covered by TNFα binding surface, steric hinderance would be present.

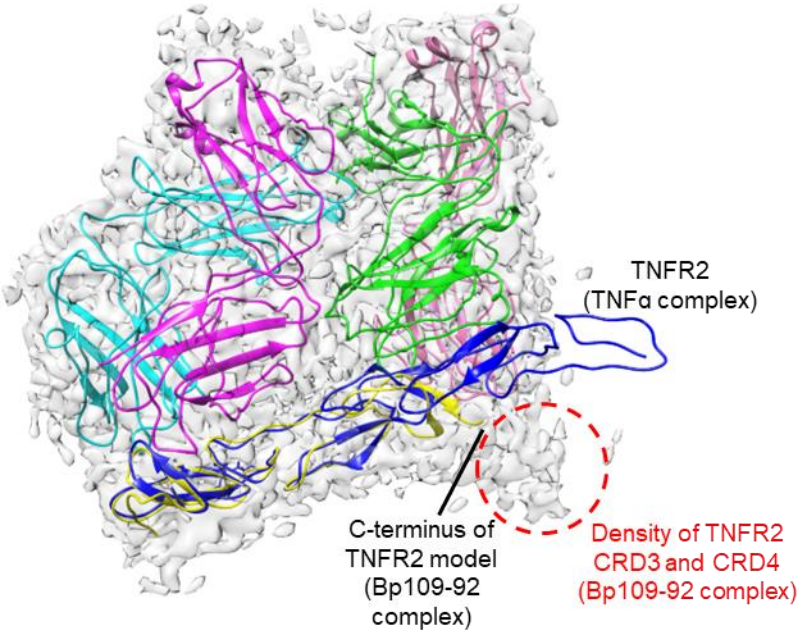


**Fig. S18. Structure comparison of Bp109-92–TNFR2-MBP complex with TNFα–TNFR2 complex by the overall view of the cryo-EM structure colored as in Fig. 4a.** The map is contoured at a lower level than Fig. 4a. TNFR2 in complex with TNFα (blue) (PDB entry: 3ALQ) is superposed with TNFR2 in complex with Bp109-92 (yellow).

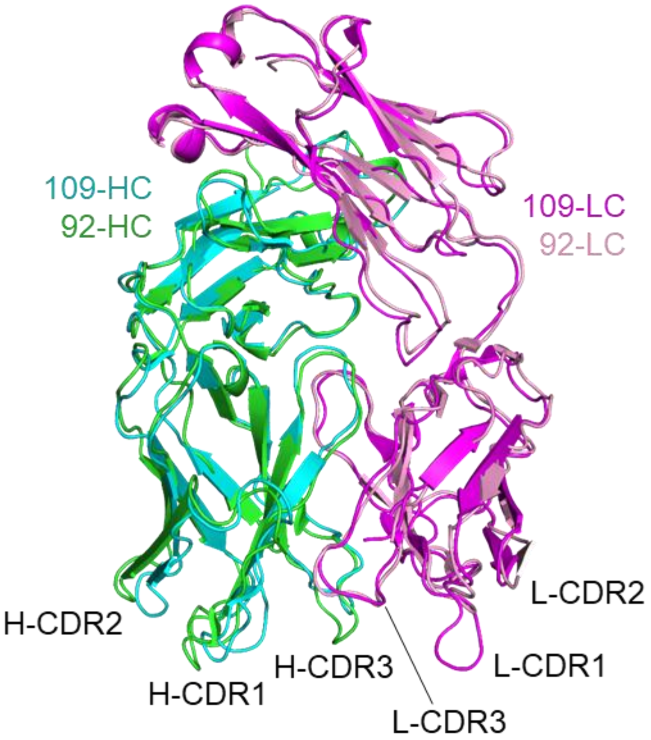


**Fig. S19. Superposition between 109-Fab and 92-Fab in the Bp109-92–TNFR2 complex.** Chains are colored as in Fig, 4a.

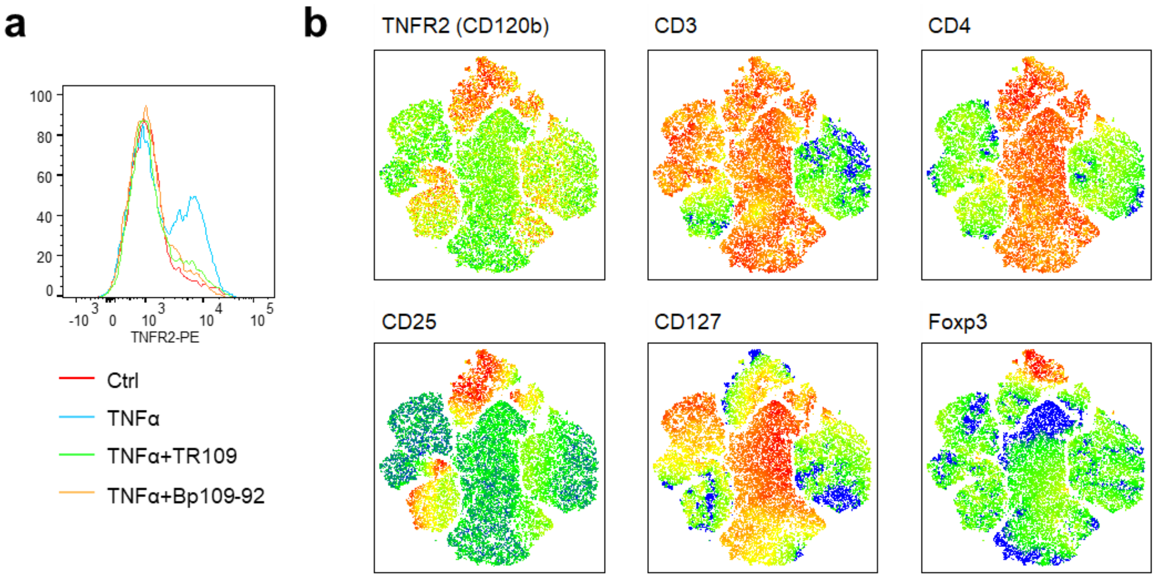


**Fig. S20. a, CD3$^+$ cells analyzed by TNFR2 expression.** Peripheral blood mononuclear cells were cultured for 48 h and the cells were analyzed using flow cytometry. Red, no stimulation (Ctrl); cyan, in the presence of 50 ng/mL TNFα (TNFα); green, in the presence of 500 ng/mL TR109 and TNFα (TNFα+TR109); orange, in the presence of 500 ng/mL Bp109-92 and TNFα (TNFα+Bp109-92). **b, TNFR2 expression in relation with several T cell markers found in cluster analysis of PBMC.** Each panel shows the expression of the labeled cellular markers. Color gradient from blue to red indicates low to high expression. For the whole population, TNFR2 expression correlated well with CD25 expression. CD3$^+$CD4$^+$TNFR2$^+$ cells were characteristic of low expression of CD127. Most Foxp3$^+$ cells were present in the TNFR2$^+$ population reflecting correlation of TNFR2 expression with CD25 and CD127.

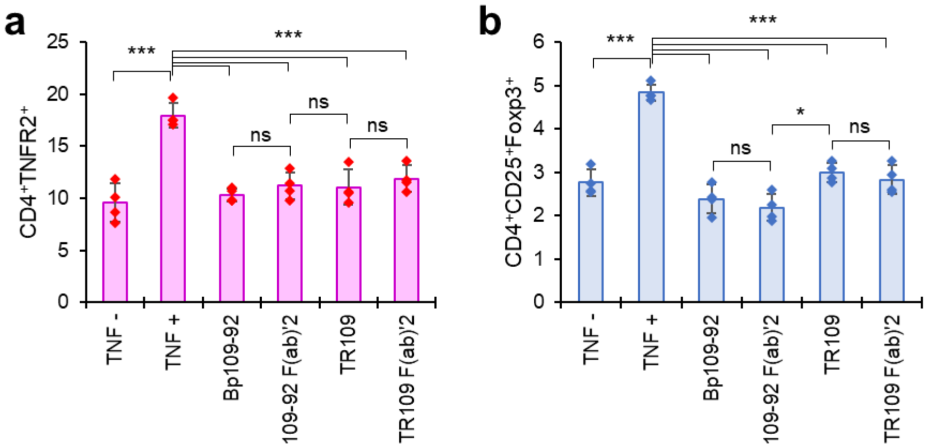


**Fig. S21. Suppression of TNFR2 activity of human T cells by the F(ab′)$_2$ of antagonists.** Population of $CD4^+TNFR2^+$ cells (**a**) or $CD4^+CD25^+Foxp3^+$ cells (**b**) among $CD3^+$ cells in PBMC without treatment (TNF−), in the presence of 50 ng/mL TNFα (TNF +), or in the presence of TNFα and antibodies. The values obtained in each experiment are shown as dots, and the average of four experiments are shown with bars. Error bars represent the standard deviations. *Statistical significance, Tukey's test, *, $P < 0.05$; ***, $P < 0.001$; ns, not significant ($P > 0.05$). See Table S4 for all adjusted $P$ values.

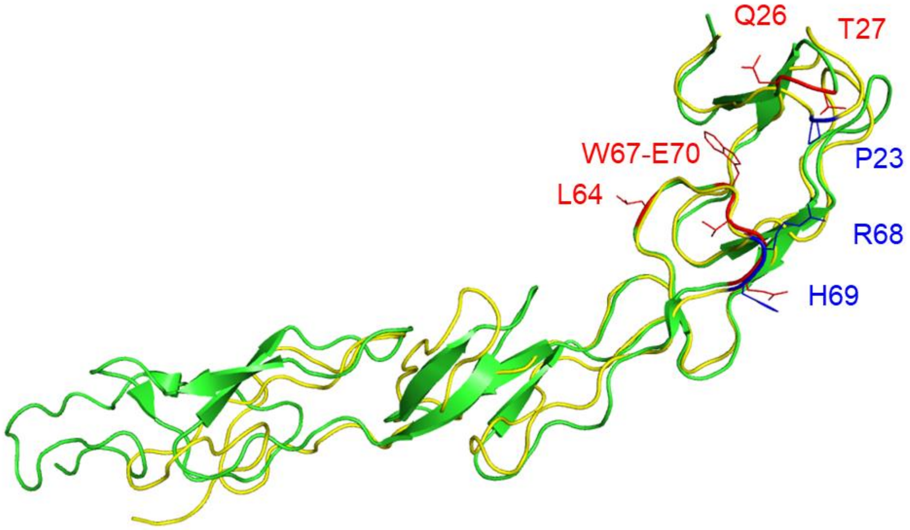


**Fig. S22. Epitopes of TR109 (anti-TNFR2 antagonist) and previously reported Atrosab (anti-TNFR1 antagonist)[30].** Atrosab is a bivalent cIgG sharing an original clone with monovalent Atrosimab[19]. Labeled amino acids were each determined by mutagenesis. Green, TNFR2 (PDB entry: 3ALQ); red, epitope of TR109, yellow, TNFR1 (PDB entry: 1TNR); blue, epitope of Atrosab[30].

**Table S1. Kinetic parameters of sTNFR2 binding to the captured antibodies[a]**

| Antibody | $k_{on}$ ($10^6$/Ms) | $k_{off}$ ($10^{-3}$/s) | $K_D$ (nM) |
|---|---|---|---|
| TR45 | 0.22 | 4.1 | 19 |
| TR92 | 0.87 | 3.5 | 4.0 |
| TR94[b] | 0.28 | 2.6 | 9.5 |
| TR96 | 0.58 | 0.24 | 0.42 |
| TR109 | 0.27 | 1.5 | 5.6 |
| Bp109-45 | 0.42 | 0.55 | 1.3 |
| Bp109-92 | 3.1 | 0.48 | 0.16 |
| Bp109-94 | 0.56 | 0.39 | 0.69 |
| Bp109-96 | 0.81 | 0.33 | 0.41 |
| Bp45-92 | 0.70 | 3.1 | 4.5 |
| Bp45-94 | 0.58 | 0.69 | 1.2 |
| Bp45-96 | 1.2 | 0.30 | 0.26 |
| Bp94-92 | 6.2 | 0.61 | 0.098 |
| Bp94-96 | 1.3 | 0.25 | 0.20 |
| Bp96-92 | 1.2 | 0.35 | 0.30 |

[a] Fitting for 1:1 binding kinetics.

[b] Parameters for TR94 are not accurate due to poor fitting.

**Table S2. Kinetic parameters of antibody binding to captured TNFR2-MBP[ab]**

| | 12RU | | | | 45RU | | | | 120RU | | | | 300RU | | | |
|---|---|---|---|---|---|---|---|---|---|---|---|---|---|---|---|---|
| Antibody | Capture (RU) | $k_{on}$ ($10^6$/Ms) | $k_{off}$ ($10^{-3}$/s) | $K_D$ (nM) | Capture (RU) | $k_{on}$ ($10^6$/Ms) | $k_{off}$ ($10^{-3}$/s) | $K_D$ (nM) | Capture (RU) | $k_{on}$ ($10^6$/Ms) | $k_{off}$ ($10^{-3}$/s) | $K_D$ (nM) | Capture (RU) | $k_{on}$ ($10^6$/Ms) | $k_{off}$ ($10^{-3}$/s) | $K_D$ (nM) |
| TR45 | 12.5 | N.D. (small response) | | | 44.6 | 1.85 | 0.0969 | 0.0523 | 115.1 | N.D. (too slow) | | | 317.4 | N.D. (too slow) | | |
| TR92 | 12.5 | 1.71 | 0.09 | 0.0527 | 44.5 | N.D. (too slow) | | | 113.3 | N.D. (too slow) | | | 325.1 | N.D. (too slow) | | |
| TR94 | 12.5 | N.D. (small response) | | | 44.8 | N.D. (small response) | | | 113.6 | 6.69 | 1.71 | 0.256 | 312.2 | 11 | 2.55 | 0.232 |
| TR96 | 12.5 | N.D. (too slow) | | | 45.2 | N.D. (too slow) | | | 112.3 | N.D. (too slow) | | | 316.5 | N.D. (too slow) | | |
| TR109 | 12.6 | 1.59 | 0.284 | 0.179 | 45.9 | 2.62 | 0.155 | 0.0589 | 115.7 | 1.88 | 0.163 | 0.0867 | 320.4 | N.D. (too slow) | | |
| Bp109-45 | 13.4 | 1.94 | 0.209 | 0.108 | 45.1 | 2.84 | 0.183 | 0.0644 | 122.4 | 1.68 | 0.088 | 0.0525 | 317.5 | 1.49 | 0.0585 | 0.0393 |
| Bp109-92 | 13.6 | 1.1 | 0.103 | 0.0938 | 45.9 | 1.61 | 0.0464 | 0.0288 | 121.3 | 1.32 | 0.0592 | 0.0447 | 302.4 | 1.01 | 0.0538 | 0.0534 |
| Bp109-94 | 13.1 | 1.82 | 0.966 | 0.531 | 45.1 | 1.98 | 0.464 | 0.235 | 121.1 | 1.49 | 0.285 | 0.191 | 320.4 | 1.33 | 0.171 | 0.129 |
| Bp109-96 | 12.8 | 0.584 | 0.217 | 0.372 | 44.6 | 1.09 | 0.0247 | 0.0227 | 117.8 | N.D. (too slow) | | | 321.3 | N.D. (too slow) | | |
| Bp45-92 | 12 | 1.09 | 0.0734 | 0.0674 | 44.8 | N.D. (too slow) | | | 118 | N.D. (too slow) | | | 317.2 | N.D. (too slow) | | |
| Bp45-94 | 11.4 | N.D. (small response) | | | 45.3 | 2.61 | 0.414 | 0.159 | 118.9 | 1.1 | 0.269 | 0.244 | 310.3 | 0.743 | 0.122 | 0.164 |
| Bp45-96 | 12.3 | 0.543 | 0.0811 | 0.149 | 44.9 | 0.884 | 0.0194 | 0.0219 | 118.9 | N.D. (too slow) | | | 319.4 | N.D. (too slow) | | |
| Bp94-92 | 12.5 | 2.49 | 0.546 | 0.219 | 46 | 2.49 | 0.238 | 0.0955 | 118.8 | 2.03 | 0.242 | 0.119 | 318.1 | 2.41 | 0.203 | 0.0842 |
| Bp94-96 | 12.5 | 0.953 | 0.0244 | 0.0256 | 45 | 1.28 | 0.103 | 0.0803 | 117.9 | 0.956 | 0.0318 | 0.0333 | 323 | 0.878 | 0.0171 | 0.0195 |
| Bp96-92 | 12.6 | 0.887 | 0.0322 | 0.0363 | 45.4 | N.D. (too slow) | | | 117.8 | N.D. (too slow) | | | 317.9 | N.D. (too slow) | | |

[a] Fitting for 1:1 binding kinetics based on single-cycle kinetics analysis.

[b] N.D., not determined (*U*-value exceeded 15) due to low binding level due to weak interaction (described as ‘small response’) or slow dissociation in the experimental condition (described as ‘too slow’).

**Table S3. Adjusted *P* values for Tukey's test for PBMC stimulation analysis (Fig. 5a,b)[a]**

| Fig. 5a CD4⁺TNFR2⁺ | | Population(%) | S.D.(%) | P value Ctrl | No Ab | Bp109-92 50 | Bp109-92 150 | Bp109-92 500 | TR109 50 | TR109 150 | TR109 500 (ng/mL) |
|---|---|---|---|---|---|---|---|---|---|---|---|
| Ctrl | | 11.79 | 1.45 | - | | | | | | | |
| No Ab | | 26.55 | 3.95 | < 0.001 | - | | | | | | |
| Bp109-92 | 50 | 22.33 | 3.06 | 0.002 | 0.565 | - | | | | | |
| Bp109-92 | 150 | 17.64 | 3.63 | 0.192 | 0.010 | 0.434 | - | | | | |
| Bp109-92 | 500 | 11.42 | 0.62 | 1 | < 0.001 | 0.001 | 0.141 | - | | | |
| TR109 | 50 | 21.08 | 2.59 | 0.007 | 0.259 | 0.999 | 0.772 | 0.004 | - | | |
| TR109 | 150 | 17.43 | 5.08 | 0.227 | 0.008 | 0.380 | 1 | 0.169 | 0.718 | - | |
| TR109 | 500 | 12.74 | 2.36 | 1 | < 0.001 | 0.005 | 0.381 | 0.999 | 0.018 | 0.435 | - |
| | (ng/mL) | | | | | | | | | | |

| Fig. 5b CD4⁺CD25⁺ Foxp3⁺ | | Population(%) | S.D.(%) | P value Ctrl | No Ab | Bp109-92 50 | Bp109-92 150 | Bp109-92 500 | TR109 50 | TR109 150 | TR109 500 (ng/mL) |
|---|---|---|---|---|---|---|---|---|---|---|---|
| Ctrl | | 3.79 | 0.53 | - | | | | | | | |
| No Ab | | 7.18 | 1.04 | 0.001 | - | | | | | | |
| Bp109-92 | 50 | 5.97 | 1.14 | 0.077 | 0.678 | - | | | | | |
| Bp109-92 | 150 | 4.86 | 1.14 | 0.789 | 0.051 | 0.752 | - | | | | |
| Bp109-92 | 500 | 3.22 | 0.30 | 0.990 | < 0.001 | 0.012 | 0.310 | - | | | |
| TR109 | 50 | 5.76 | 0.86 | 0.140 | 0.492 | 1 | 0.895 | 0.024 | - | | |
| TR109 | 150 | 5.46 | 1.44 | 0.298 | 0.262 | 0.995 | 0.988 | 0.064 | 1 | - | |
| TR109 | 500 | 4.08 | 0.99 | 1 | 0.004 | 0.172 | 0.949 | 0.912 | 0.288 | 0.526 | - |
| | (ng/mL) | | | | | | | | | | |

[a] Adjusted *P* values no less than 1 are shown as 1.

**Table S4. Adjusted *P* values for Tukey's test for PBMC stimulation analysis (Fig. S21)[a]**

| Fig. S21a CD4⁺TNFR2⁺ | Population(%) | S.D.(%) | P value | | | | | |
|---|---|---|---|---|---|---|---|---|
| | | | TNF − | TNF + | Bp109-92 | 109-92 F(ab')2 | TR109 | TR109 F(ab')2 |
| TNF − | 9.56 | 1.84 | - | | | | | |
| TNF + | 17.95 | 1.17 | < 0.001 | - | | | | |
| Bp109-92 | 10.31 | 0.65 | 0.969 | < 0.001 | - | | | |
| 109-92 F(ab')2 | 11.20 | 1.32 | 0.557 | < 0.001 | 0.937 | - | | |
| TR109 | 11.05 | 1.69 | 0.651 | < 0.001 | 0.971 | 1 | - | |
| TR109 F(ab')2 | 11.89 | 1.25 | 0.208 | < 0.001 | 0.592 | 0.978 | 0.949 | - |

| Fig. S21b CD4⁺CD25⁺ Foxp3⁺ | Population(%) | S.D.(%) | P value | | | | | |
|---|---|---|---|---|---|---|---|---|
| | | | TNF − | TNF + | Bp109-92 | 109-92 F(ab')2 | TR109 | TR109 F(ab')2 |
| TNF − | 2.76 | 0.31 | - | | | | | |
| TNF + | 4.83 | 0.19 | < 0.001 | - | | | | |
| Bp109-92 | 2.38 | 0.34 | 0.449 | < 0.001 | - | | | |
| 109-92 F(ab')2 | 2.19 | 0.32 | 0.099 | < 0.001 | 0.927 | - | | |
| TR109 | 3.00 | 0.23 | 0.863 | < 0.001 | 0.071 | 0.010 | - | |
| TR109 F(ab')2 | 2.83 | 0.33 | 1 | < 0.001 | 0.289 | 0.054 | 0.962 | - |

[a] Adjusted *P* values no less than 1 are shown as 1.
