# Supplementary material for: Development of a 1:1-binding biparatopic anti-TNFR2 antagonist by reducing signaling activity through epitope selection
Source: Commun Biol. 2023 Sep 27;6:987. doi: 10.1038/s42003-023-05326-8 (PMC10533564; doi:10.1038/s42003-023-05326-8)
Supplement: Supplementary file 2 — Description of Additional Supplementary Files [file 42003_2023_5326_MOESM2_ESM.pdf]

Description of Additional Supplementary Files

File name: Supplementary Data 1
Description: Sequences of the mutants used for the epitope identification.

File name: Supplementary Data 2
Description: The source data behind the graphs in the paper.
