# Supplementary material for: Development of a 1:1-binding biparatopic anti-TNFR2 antagonist by reducing signaling activity through epitope selection
Source: Commun Biol. 2023 Sep 27;6:987. doi: 10.1038/s42003-023-05326-8 (PMC10533564; doi:10.1038/s42003-023-05326-8)
Supplement: Supplementary file 5 — Reporting Summary [file 42003_2023_5326_MOESM5_ESM.pdf]

nature portfolio

Corresponding author(s): Hiroki Akiba, Satoshi Nagata and Kouhei Tsumoto

Last updated by author(s): Aug 11, 2023

Reporting Summary

Nature Portfolio wishes to improve the reproducibility of the work that we publish. This form provides structure for consistency and transparency in reporting. For further information on Nature Portfolio policies, see our Editorial Policies and the Editorial Policy Checklist.

Statistics

For all statistical analyses, confirm that the following items are present in the figure legend, table legend, main text, or Methods section.

n/a	Confirmed	
☐	☒	The exact sample size (*n*) for each experimental group/condition, given as a discrete number and unit of measurement
☐	☒	A statement on whether measurements were taken from distinct samples or whether the same sample was measured repeatedly
☐	☒	The statistical test(s) used AND whether they are one- or two-sided<br>*Only common tests should be described solely by name; describe more complex techniques in the Methods section.*
☒	☐	A description of all covariates tested
☒	☐	A description of any assumptions or corrections, such as tests of normality and adjustment for multiple comparisons
☐	☒	A full description of the statistical parameters including central tendency (e.g. means) or other basic estimates (e.g. regression coefficient) AND variation (e.g. standard deviation) or associated estimates of uncertainty (e.g. confidence intervals)
☐	☒	For null hypothesis testing, the test statistic (e.g. *F*, *t*, *r*) with confidence intervals, effect sizes, degrees of freedom and *P* value noted<br>*Give P values as exact values whenever suitable.*
☒	☐	For Bayesian analysis, information on the choice of priors and Markov chain Monte Carlo settings
☒	☐	For hierarchical and complex designs, identification of the appropriate level for tests and full reporting of outcomes
☒	☐	Estimates of effect sizes (e.g. Cohen's *d*, Pearson's *r*), indicating how they were calculated

Our web collection on statistics for biologists contains articles on many of the points above.

Software and code

Policy information about availability of computer code

Data collection	EnSpire multimode reader v4.0 (PerkinElmer), FACSDiva v8.0.1 (BD), Biacore T200 control software 2.0.1 or 3.2.1 (Cytiva), UNICORN (Cytiva), ASTRA 6.0 (Wyatt), Refeyn One (Refeyn); Cryo-EM, SerialEM v3.8, yoneoLocr v1.0. Negative staining, EMMENU v4.0
Data analysis	Flow cytometry, FACSDiva v8.0.1; FlowJo v10.8.1; SPR, Biacore Evaluation Software v2.0 or v3.2.1; SEC-MALS, ASTRA 6.0; Cryo-EM, RELION v4.0; cryoSPARC v3.3.2; SWISS-MODEL; UCSF Chimera v1.15; Coot v0.9.6; PHENIX v1.19.2; PyMOL v2.5.0., Negative staining, RELION v2.0; Microsoft Excel 2019; R 4.3.0

For manuscripts utilizing custom algorithms or software that are central to the research but not yet described in published literature, software must be made available to editors and reviewers. We strongly encourage code deposition in a community repository (e.g. GitHub). See the Nature Portfolio guidelines for submitting code & software for further information.

Data

Policy information about availability of data

All manuscripts must include a data availability statement. This statement should provide the following information, where applicable:

- Accession codes, unique identifiers, or web links for publicly available datasets
- A description of any restrictions on data availability
- For clinical datasets or third party data, please ensure that the statement adheres to our policy

Cryo-EM density map and model of Bp109-92 in complex with TNFR2-MBP are deposited to Electron microscopy Data Bank (EMDB) and Protein Data Bank (PDB) with accession codes of EMD-34871 and PDB-8HLB, respectively. All other data are available in the main text or supplementary materials.

Human research participants

Policy information about studies involving human research participants and Sex and Gender in Research.

Reporting on sex and gender	*Use the terms sex (biological attribute) and gender (shaped by social and cultural circumstances) carefully in order to avoid confusing both terms. Indicate if findings apply to only one sex or gender; describe whether sex and gender were considered in study design whether sex and/or gender was determined based on self-reporting or assigned and methods used. Provide in the source data disaggregated sex and gender data where this information has been collected, and consent has been obtained for sharing of individual-level data; provide overall numbers in this Reporting Summary. Please state if this information has not been collected. Report sex- and gender-based analyses where performed, justify reasons for lack of sex- and gender-based analysis.*
Population characteristics	*Describe the covariate-relevant population characteristics of the human research participants (e.g. age, genotypic information, past and current diagnosis and treatment categories). If you filled out the behavioural & social sciences study design questions and have nothing to add here, write "See above."*
Recruitment	*Describe how participants were recruited. Outline any potential self-selection bias or other biases that may be present and how these are likely to impact results.*
Ethics oversight	*Identify the organization(s) that approved the study protocol.*

Note that full information on the approval of the study protocol must also be provided in the manuscript.

Field-specific reporting

Please select the one below that is the best fit for your research. If you are not sure, read the appropriate sections before making your selection.

☒ Life sciences ☐ Behavioural & social sciences ☐ Ecological, evolutionary & environmental sciences

For a reference copy of the document with all sections, see nature.com/documents/nr-reporting-summary-flat.pdf

Life sciences study design

All studies must disclose on these points even when the disclosure is negative.

Sample size	All data shown are representative of at least three independent experiments except for binding experiment in Fig. S7a which was conducted only twice. No statistical method was used to predetermine the sample sizes. For cryo-EM image processing, 2,218,071 particle images were picked from 4,160 micrographs, and 100,391 particle images were used for final reconstruction.
Data exclusions	Data exclusion was conducted for flow cytometry analysis using PBMC to standardize the number of cells analyzed among conditions. Exclusion was randomly performed using DownSample plugin in FlowJo software. For cryo-EM image processing, 2,117,680 particle images were excluded from the initial 2,218,071 particle images.
Replication	All data shown are representative of at least three independent replicate experiments except for binding experiment in Fig. S7a which was conducted only twice. It was clearly stated in the manuscript whether the findings are based on a single experiment or if the results have been replicated in multiple experiments. For the cryo-EM study, two grids were prepared to obtain similar results, and the structural analysis was performed from a single dataset.
Randomization	Not relevant because all biological experiments were conducted by separating cells from a single source.
Blinding	Not relevant because all the in vitro experiments performed in this study do not require blinding of the subjects, investigators, or assessors of the outcome measures.

Reporting for specific materials, systems and methods

We require information from authors about some types of materials, experimental systems and methods used in many studies. Here, indicate whether each material, system or method listed is relevant to your study. If you are not sure if a list item applies to your research, read the appropriate section before selecting a response.

Materials & experimental systems

n/a	Involved in the study
☐	☒ Antibodies
☐	☒ Eukaryotic cell lines
☒	☐ Palaeontology and archaeology
☒	☐ Animals and other organisms
☒	☐ Clinical data
☒	☐ Dual use research of concern

Methods

n/a	Involved in the study
☒	☐ ChIP-seq
☐	☒ Flow cytometry
☒	☐ MRI-based neuroimaging

Antibodies

Antibodies used

Anti-TNFR2 monoclonal antibodies TR45, TR92, TR94, TR96 and TR109 were originally developed and are described in a patent application WO/2018/192907.
Anti-TNFR2 human IgG1-chimeric and biparatopic antibodies were developed for this study and are described in this article. Sequences of the variable regions are disclosed in a patent application WO/2021/200840.
Anti-mouse IgG, Fcgamma-specific, Jackson ImmunoResearch, Cat. No. 115-005-071, coating 2ug/ml
ALP Anti-rabbit IgG, Fcgamma-specific, Jackson ImmunoResearch, Cat. No. 111-005-046, Dilution 1:4000
PE Anti-human IgG, Fcgamma-specific, Jackson ImmunoResearch, Cat. No. 109-116-170, dilution 1:200
BV711 human CD3, BioLegend, Cat. No. 300463 (clone UCHT1), lot B317979, dilution 1:250
BV510 human CD4, BioLegend, Cat. No. 344633 (clone SK3), lot B310865, dilution 1:100
BV421 human CD25, BioLegend, Cat. No. 302629 (clone BC96), lot B311393, dilution 1:40
BV605 human CD25, BioLegend, Cat. No. 302631 (clone BC96), lot B280080, dilution 1:40
PE human TNFR2 (CD120b), R&D Systems, Cat. No. 22235 (clone FAB226P), lot LFB0713081, dilution 1:70 (Fig. S6) or 1:100 (other)
PE Mouse IgG2a, kappa, isotype control, BioLegend, Cat. No. 400212 (clone MOPC-173), lot B376292, dilution 1:70
PerCP-Cy5.5 human CD127, BD Biosciences, Cat. No. 560551 (clone HIL-7R-M21), lot 1046632, dilution 1:40
AlexaFluor 647 human Foxp3, BioLegend, Cat. No. 320213 (clone 259D), lot B290108, dilution 1:100
Anti-human IgG (Fc), part of Human antibody capture kit, Cytiva, Cat. No. BR100839, immobilized to ca. 10000 RU
Anti-MBP Monoclonal Antibody, New England BioLabs, Cat. No. E8032S, immobilized to ca. 6000 RU

Validation

For antibodies expressed recombinantly in-house, the concentration of purified IgGs was determined by absorbance at 280 nm. Quality assessment of purified antibodies was performed by SDS-PAGE and binding assays as described in this manuscript.
For immunochemical reagents, PE-human IgG, BV711-CD3, BV510-CD4, BV421-CD25, PE-TNFR2, PerCP-Cy5.5-CD127, AlexaFluor647-Foxp3 were validated by the manufacturer for use in flow cytometry.
We verified that anti-human TNFR2 clone FAB226P did not compete with TNF-alpha, TR109 or TR92.

Eukaryotic cell lines

Policy information about cell lines and Sex and Gender in Research

Cell line source(s)	Expi293F, HEK293T, Ramos-Blue, and Ramos-Blue stably transfected with TNFR2
Authentication	Expi293F (ThermoFisher), HEK293T (ATCC), and Ramos-Blue (InVivogen) cells were authenticated by the provider. TNFR2 transfectant was developed in our laboratory and not authenticated.
Mycoplasma contamination	Every 6 months, we routinely check for mycoplasma contamination of cultured cells in our laboratory by using MycoAlert™ Mycoplasma Detection Kit (Lonza, LT07-318). We confirm that the cell lines used in this study were negative for mycoplasma contamination.
Commonly misidentified lines (See ICLAC register)	Not used in this study.

Flow Cytometry

Plots

Confirm that:

☒ The axis labels state the marker and fluorochrome used (e.g. CD4-FITC).

☒ The axis scales are clearly visible. Include numbers along axes only for bottom left plot of group (a 'group' is an analysis of identical markers).

☒ All plots are contour plots with outliers or pseudocolor plots.

☐ A numerical value for number of cells or percentage (with statistics) is provided.

Methodology

Sample preparation	HEK293T cells were detached from the culture dish using 0.25% trypsin - 1 mM EDTA prior to staining. Other cells were stained as cultured. Cells were treated with PBS containing 0.2% sodium azide and 5% FBS. For antibody-binding analysis of TNFR2-expressing Ramos-Blue and HEK293T cells, primary antibodies were first incubated in a dilution series for 30 min on ice and then labeled with PE anti-human IgG. For PBMC, membrane proteins were stained in the same protocol as above, and the cells were fixed with eBioscience Intracellular Fixation & Permeabilization Buffer Set (Thermo). AlexaFluor647-Foxp3 was incubated in the 1x Permeabilization buffer containing 2% FBS. The cells were passed through 40 micrometer filter prior to flow cytometry analysis.
Instrument	LSRFortessa flow cytometer X-20 (BD Biosciences)
Software	FACSDiva v8.0.1; FlowJo v10.8.1
Cell population abundance	Not applicable because sorting was not conducted.
Gating strategy	For antibody-binding analysis of TNFR2-expressing Ramos-Blue and HEK293T cells, preliminary FSC/SSC gating was conducted. For PBMC analysis, dead cells stained by LIVE/DEAD™ Fixable Near IR (780) Viability Kit (Thermo Fisher Scientific) was first excluded by Near-IR/FSC gating, then doublets were excluded by FSC-A/FSC-H, and FSC/SSC gating followed (Supplementary Fig. S5).

☒ Tick this box to confirm that a figure exemplifying the gating strategy is provided in the Supplementary Information.
